# Numerical investigation of nanoparticles slip mechanisms impact on the natural convection heat transfer characteristics of nanofluids in an enclosure

**DOI:** 10.1038/s41598-021-95269-z

**Published:** 2021-08-03

**Authors:** Muritala Alade Amidu, Yacine Addad, Mohamed Kamel Riahi, Eiyad Abu-Nada

**Affiliations:** 1 Department of Nuclear Engineering, Khalifa University of Science and Technology, Abu Dhabi, United Arab Emirates; 2 Emirates Nuclear Technology Center (ENTC), Khalifa University of Science and Technology, Abu Dhabi, United Arab Emirates; 3 Department of Mathematics, Khalifa University of Science and Technology, Abu Dhabi, United Arab Emirates; 4 Department of Mechanical Engineering, Khalifa University of Science and Technology, Abu Dhabi, United Arab Emirates

**Keywords:** Engineering, Mechanical engineering

## Abstract

This study intends to give qualitative results toward the understanding of different slip mechanisms impact on the natural heat transfer performance of nanofluids. The slip mechanisms considered in this study are Brownian diffusion, thermophoretic diffusion, and sedimentation. This study compares three different Eulerian nanofluid models; Single-phase, two-phase, and a third model that consists of incorporating the three slip mechanisms in a two-phase drift-flux. These slip mechanisms are found to have different impacts depending on the nanoparticle concentration, where this effect ranges from negligible to dominant. It has been reported experimentally in the literature that, with high nanoparticle volume fraction the heat transfer deteriorates. Admittingly, classical nanofluid models are known to underpredict this impairment. To address this discrepancy, this study focuses on the effect of thermophoretic diffusion and sedimentation outcome as these two mechanisms turn out to be influencing players in the resulting heat transfer rate using the two-phase model. In particular, the necessity to account for the sedimentation contribution toward qualitative modeling of the heat transfer is highlighted. To this end, correlations relating the thermophoretic and sedimentation coefficients to the nanofluid concentration and Rayleigh number are proposed in this study. Numerical experiments are presented to show the effectiveness of the proposed two-phase model in approaching the experimental data, for the full range of Rayleigh number in the laminar flow regime and for nanoparticles concentration of (0% to 3%), with great satisfaction.

## Introduction

Nanofluid technology is being considered in many engineering applications and represents one of the most attractive heat transfer research areas nowadays. Benefits from this technology, for various industries, have been discussed thoroughly in the literature. The nanofluid preparation consists of dispersing one of; metallic, ceramic, Polymer nanotube, and Carbon nano-sized powder in a base fluid. Admittedly, the heat transfer performance of the resulting nanofluid is highly dependent on the thermophysical properties of the mixture and the dispersion of the nanoparticles within the studied system.

As a subtopic of nanofluid research activities, the buoyancy-induced convection of nanofluids in enclosures has been profusely studied both experimentally and numerically over the last twenty years. Previous experimental studies by researchers using different nanoparticle materials, such as; Al_2_O_3_, CuO, and TiO_2_ (Putra et al*.*^1^, Nnanna^2^, Chang et al*.*^3^, Ho et al*.*^4^, and Hu et al*.*^5^) have shown a unanimous finding that heat transfer gets deteriorated by nanofluid contained within a differentially heated enclosure in natural convection applications. Putra et al*.*^1^ for instance, attributed this decrease to result from the particle/fluid slip and sedimentation of nanoparticles. In numerical simulation, however, opinions are divided concerning the best way to capture this experimentally observed nanofluid heat transfer deterioration.

A few methods have been proposed in the literature to model nanofluids and associated heat transfer. These methods range from; methods at the molecular level, i.e., using molecular dynamic simulations (see for example the work of Keblinski et al*.*^6^ and Cui et al*.*^7^ amongst others) and nanoscale level models using Boltzmann transport equation (e.g., the study conducted by Sheikholeslami et al*.*^8^) to methods based on continuum mechanics. The latter can be also subdivided into; single-phase approach, two-phase approach based on the Eulerian–Lagrangian method (see for example the work of Sharaf et al*.*^9^), or two-phase approach based on the Eulerian-Eulerian method, which is the focus of the present study. Considering this, a brief review of the evolution of Eulerian-Eulerian based models used for the prediction of nanofluid heat transfer features in the square cavity is presented here.

At the nascent stage of numerical methods development for nanofluid heat transfer, the single-phase modeling approach took the center stage mainly due to its simplicity, even though the nanofluid heat transfer process is a multiphase problem. Quite many researchers have performed numerical investigations of the natural convection heat transfer of nanofluid using a single-phase model. A non-exhaustive list of studies reported in the open literature comprises; square cavities with straight or wavy walls, cylindrical cavities, cavities of different shapes with a heat source, and so forth. However, for consistency with the scope of the present study, the review here is limited to the differentially heated quadrate enclosure problems. Several characteristics of natural convection in a quadrate enclosure such as; effects of Rayleigh number (or Grashof number), nanoparticles concentration, the enclosure inclination angle, heating methods, cavity size, and varying thermophysical properties have been investigated by many authors using the single-phase model. In this model, the nanofluid is treated as a single-phase fluid but with bulk nanofluid thermophysical properties. Thus, the basic assumption beneath the single-phase approach is that nanoparticles are homogeneously distributed in the base fluid and therefore can be assumed as a single fluid. Khanafer et al*.*^10^ used the single-phase model to study nanofluid heat transfer enhancement in a two-dimensional enclosure considering the solid nanoparticles dispersion. In that pioneering work, the model was used to analyze the impact of the nanoparticles on the fluid flow and heat transfer processes within the enclosure. The paper had a few flaws including the consideration of very high nanoparticles concentration (up to 25%) without accounting neither for agglomeration effects nor for the nanofluid stability (from a practical point of view), model validation using only commercial code data for pure fluid and so on. Nevertheless, this might be understandable by admitting the sparsity of experimental data and the little information available to the research society to work with then. Certainly, the authors were able to identify the fact that the thermophysical properties expressions, used for the nanofluid, significantly affected the results. In the same vein, Abu-Nada and Oztop^11^ adopted a single-phase model to study the effects of inclination angle on natural convection in enclosures filled with Cu-water nanofluid. The angle of inclination served as the control parameter for fluid flow and heat transfer processes. Also, Aminossadati and Ghasemi^12^ investigated the influences of; Rayleigh number, location and geometry of the heat source, and volume fraction of nanoparticles on the natural convection cooling of a heat source placed at the bottom wall of an enclosure filled with nanofluid. With the same single-phase modeling approach, Oztop and Abu-Nada^13^ studied the buoyancy-driven nanofluid heat transfer in a partially filled enclosure using different kinds of nanoparticles where it was found that the location of the heater has a significant impact on the nanofluid heat transfer and the associated fluid flow. More recently, Ibrahim et al*.*^14^ performed a comprehensive study to demonstrate how the loading of nanoparticles could reduce the positive effect on thermal conductivity.

Considering the above, it could be deduced that some of the authors of previous studies were able to qualitatively predict the observed deterioration of the nanofluid heat transfer but the accuracies of their predictions were not verified with experimental data as pointed out by Chen et al*.*^15^ even though the investigated characteristics (Rayleigh number, inclination angle, and so forth) might still be valid in terms of trends. Therefore, it might be premature to conclude that the single-phase model is adequate for the prediction of nanofluid thermal behavior based on these studies. To buttress the doubt concerning the accuracy of the single-phase model’s prediction of nanofluid thermal behavior; an experimental study performed by Ho et al*.*^4^ has indicated that the observed variable thermos-physical properties are insufficient to explain the heat transfer behavior of nanofluid at high concentrations.

To transcend the limitations, stated above, of the single-phase model, some researchers have explored alternative models that can capture the actual physics of the nanofluid thermal behavior with better explanations for the deterioration of heat transfer previously observed in experimental studies at high nanoparticle concentrations. For instance, Aminfar and Haghgoo^16^ have recently provided an interpretation of this heat transfer degradation by suggesting that the slip motion occurring between the nanoparticles and base fluid could lead to the formation of stagnant thin layers of settled nanoparticles at the bottom adiabatic wall and pure base fluid at the top adiabatic wall. This was claimed to cause a significant deterioration of the heat transfer across the enclosure. Thus, a mechanistic way to capture the nanofluid heat transfer deterioration behavior is to consider the relative motion of nanoparticles to the base fluid. This could result in non-uniform distribution of nanoparticles which in its turn, could have a significant impact on the energy and momentum transfer within the nanofluid. To actualize this, the use of a two-phase model then becomes imperative since the nanoparticles' slip motions cannot be captured with the classical single-phase model.

Different researchers have used different two-phase modeling approaches to capture various slip mechanisms they deemed dominant. For instance, a two-phase mixture model that considers nanoparticle slip due to only resistance to flow by particles (drag force) has been used by Chen et al*.*^15^ in which a reasonable agreement with experimental data was obtained. Moreover, a two-phase mixture model that considers sedimentation (i.e., gravity-driven movement) of nanoparticles as the sole slip mechanism has been used by Meng et al*.*^17^. Unfortunately, the accuracy of the model was not reported as its’ prediction was not compared with experimental data. Moreover, based on the strong model put forth by Buongiorno^18^ concerning the effect of nanoparticles Brownian diffusion and thermophoretic diffusion, a two-component model accounting for the influence of Brownian diffusion and thermophoretic diffusion has been used by Haddad et al*.*^19^ (although without validation against experimental data) and by Corcione et al*.*^20^ who reported a validation to experimental data within ± 10% error margin where the effect of slip mechanisms in the momentum and energy equations were not considered in their model.

The few previous two-phase studies that considered nanoparticle slip mechanisms especially thermophoretic diffusion term, typically relied on the McNab-Meisen^21^ model for the calculation of the thermophoretic coefficient. Interestingly, this model (McNab-Meisen relation) has been reported by Giddings et al*.*^22^ to substantially underestimate the contribution of thermophoretic diffusion to the nanoparticle migration phenomenon. Therefore, to address the shortcoming of the McNab-Meisen relation, Corcione et al*.*^20^ have attempted to leverage experimental data to develop an empirical correlation for the prediction of the thermophoretic diffusion coefficient. However, their correlation only depends on the nanoparticle concentration and does not take into account the Rayleigh number effects. More so, a detailed review of the control parameters of natural convection in differently shaped cavities with nanofluid recently performed by Rostami et al*.*^23^ has revealed that previous two-phase analysis of nanofluid heat transfer could still not predict accurate data consistent with experimental observations. Besides, a clear-cut impact of the nanoparticle slip mechanisms has not been presented in contrast with the experimental data. Hence, further studies of nanoparticle deposition in terms of model development for the various nanoparticle transport phenomena have been encouraged.

Considering this, the three key slip mechanisms (sedimentation, Brownian diffusion, and thermophoretic diffusion) are incorporated into the two-phase drift flux model. The aim here is to perform an elaborate investigation of the impact of these slip mechanisms on the thermal behavior of nanofluid in a quadrate enclosure. Prior to assessing these nanoparticle slip motions; an evaluation of existing single-phase and two-phase models is performed to ascertain the findings of the literature review. Furthermore, robust empirical correlations for the thermophoretic and sedimentation coefficients are proposed to improve the prediction of the nanoparticle migration due to both; thermophoretic diffusion and sedimentation. The correlations proposed here account, not only, for the nanoparticle concentration but also take into account the Rayleigh number variation effects.

## Thermophysical properties of nanofluid

The thermal conductivity of the base fluid gets enhanced by the suspended conductive nanoparticles such as Al_2_O_3_ with a diameter of less than 100 nm but this could also cause an undesirable increase in fluid viscosity as explained by Das et al*.*^24^. The thermophysical properties of the base fluid (water) and alumina nanoparticles which are extracted from the article of Ho et al*.*^4^ are summarized in Table 1. Over the years, several correlations have been suggested to generalize the estimation of the thermophysical properties such as thermal conductivity, specific heat capacity, viscosity, and density. However, some of these correlations were developed from specific experimental data which implies that they may be limited by the corresponding experimental conditions. For instance, Ghanbarpour et al*.*^25^ found that their experimental data of nanofluid thermal conductivity and viscosity were under-predicted by previous correlations of Maxwell^26^ and Einstein^27^ and this was also corroborated by Albojamal and Vafai^28^. Therefore, due to lack of generalization, care must be taken concerning an acceptable range of validity when selecting appropriate correlations for calculating the nanofluid thermal conductivity and viscosity.

**Table 1:** Thermophysical properties of water and alumina nanoparticles at T = 293 K by Ho et al*.*^4^.

Properties	Water	Alumina nanoparticles (Al_2_O_3_)
Specific heat capacity, ${C}_{p}$, J/kgK	4182	765
Thermal conductivity, $k$, W/mK	0.6	30
Density, $\rho$, kg/m^3^	998.2	3600
Dynamic viscosity, $\mu$, kg/ms	0.0001003	–
Nanoparticle diameter, ${d}_{p}$, nm	–	33
Volumetric expansion coefficient, $\beta$, K^1^	210 × 10^–6^	4.86 × 10^–6^

The thermophysical properties of the resulting nanofluid can be derived from the thermophysical properties of the individual components (Table 1). From the literature, two methods are commonly used to calculate these thermophysical properties. The first one assumes that the properties are only dependent on the volume fraction ($\varphi$) of the nanoparticles. The second method is more general in the sense that the physical properties are said to also depend on the temperature of the nanofluid as reported by Nguyen et al*.*^29^. Typical correlations for calculating temperature-dependent viscosity and thermal conductivity are reported in the work of Abu-Nada^30^, Abu-Nada and Chamkha^31^, Khanafer and Vafai^32^, Bianco et al*.*^33^, and Palm et al*.*^34^. It is worth mentioning, however, that dependence of the nanofluids thermophysical properties on the base fluid pH, the surfactant type, and surfactant concentration has been also reported in the literature (see Yoo et al*.*^35^ and Das et al*.*^36^). However, to avoid the uncertainty that could result from such temperature and/or stabilizer dependencies, the relations provided in the paper of Ho et al*.*^4^ (reference data in this study) are used and these correlations are summarized in Table 2. The exclusion of the physical properties dependence on temperature is further justified by the fact that a low-temperature difference of 1 K is used in the differential heated enclosure considered herein.

**Table 2:** Empirical correlations for calculating the nanofluid thermophysical properties based on the experimental data of Ho et al*.*^4^.

Properties	Volume-fraction dependent	Root mean square deviation
Dynamic viscosity	$\frac{{\mu }_{m}}{{\mu }_{bf}}=1+4.97\varphi +222.4{\varphi }^{2}$	0.024393
Thermal conductivity	$\frac{{k}_{m}}{{k}_{bf}}=1+2.72\varphi +4.97{\varphi }^{2}$	0.02069
Volumetric expansion coefficient	${{\beta }_{m}=(\left(1-\varphi \right){\rho }_{bf}{\beta }_{bf}+\varphi {\rho }_{p}{\beta }_{p})/}_{{\rho }_{m}}$	0.033401
Density	${\rho }_{m}=\left(1-\varphi \right){\rho }_{bf}+\varphi {\rho }_{p}$	0.002291
Specific heat capacity	${C}_{pm}=(\left(1-\varphi \right){\rho }_{bf}{C}_{pbf}+\varphi {{\rho }_{p}C}_{pp})/{\rho }_{m}$	0.033401

## Mathematical model

The natural convection flow considered in this study is presumed to be laminar as the Rayleigh number is less than 7 × 10^6^ and three modeling approaches are employed in the simulations. These modeling approaches are; the single-phase model, and two variants of the two-phase modeling approach (the mixture model and the two-component non-homogeneous model). Detailed mathematical representations of these modeling approaches are provided in the following sub-sections.

### Single-phase model governing equations

The single-phase modeling approach is based on the assumption that the nanoparticles are uniformly distributed in the base fluid such that the nanofluid can be treated as a single-phase fluid. An existing transient solver for buoyant-driven natural convection flow in the open-source code OpenFOAM version 6 named “buoyantBoussinesqPimpleFoam” is employed in this study to capture the nanofluid heat transfer behavior. Aside from the incorporation of variable thermo-physical properties, this solver needs no further modification. The basic assumptions of the single-phase model are that there is no slip velocity between the particles and the base fluid, the nanosuspensions and the base fluid are in local thermal equilibrium, and the thermophysical properties are dependent only on the average nanoparticle concentration. The governing equations of this solver include conservation equations for mass, momentum, and energy as given by Eqs. (1), (2), and (3), respectively.

1 $$\nabla \cdot u=0$$

2 $$\frac{\partial u}{\partial t}+\nabla \cdot \left(uu\right)=-\nabla \left(\frac{P}{{\rho }_{m}}\right)+\nabla \cdot \left(\frac{{\mu }_{m}}{{\rho }_{m}}\nabla u\right)+{g}_{k}$$

3 $$\frac{\partial T}{\partial t}+\nabla \cdot \left(Tu\right)=\nabla \cdot \left(\frac{{k}_{m}}{{\rho }_{m}{C}_{pm}}\nabla T\right)$$

The Boussinesq gravity term ${g}_{k}$ in Eq. (2) is computed using the Boussinesq approximation given by Eq. (4) where it is assumed that density variations are small to extent that they have no effects on the flow field except that they give rise to buoyancy force. Finally, the nanofluid is assumed to be incompressible.

4 $${g}_{k}=\left[1-{\beta }_{m}\left(T-{T}_{ref}\right)\right]g$$

Here $u$, $t$, $T$, $P$, and $g$ represent the velocity vector, time, temperature pressure, and gravity vector respectively. Additionally, the physical properties of the nanofluid: dynamic viscosity, density, specific heat capacity, thermal conductivity, and volumetric expansion coefficient are represented by ${\mu }_{m}$, ${\rho }_{m}$, ${C}_{pm}$, ${k}_{m}$, and ${\beta }_{m}$ and are defined in Table 2.

### Mixture model governing equations

Arguably, the non-uniformity of nanoparticle concentration in the quadrate enclosure disqualifies the use of a single-phase model for the prediction of the nanofluid heat transfer. This is since the basic single-phase model assumption (homogenous distribution of nanoparticles) no longer holds. Thus, one way to investigate the potential effect of nanoparticles' non-uniformity is to employ a mixture modeling approach. In mixture modeling approaches, aside from non-uniform nanoparticle distribution, other assumptions of the single-phase model still hold. In addition, the slip motion of nanoparticles caused by the gravity force is considered while thermophysical properties of the nanofluid are evaluated based on the local concentration of the nanoparticles.

**Figure 1:**
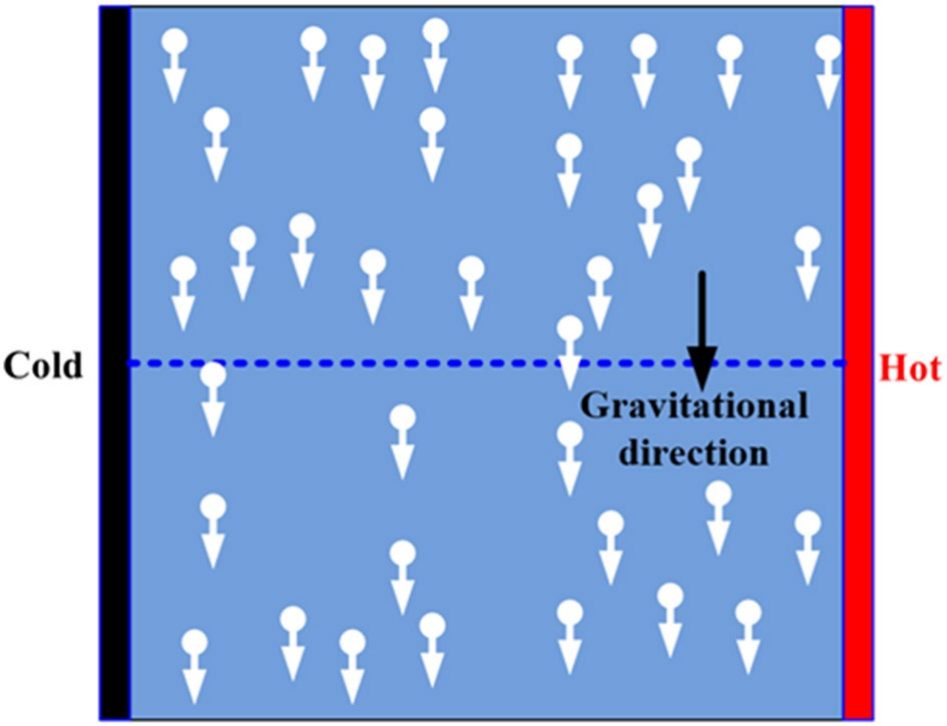
Illustration of nanoparticle sedimentation in a quadrate enclosure.

The gravity force is considered in the mixture model based on the previous experimental observation by Chang et al*.*^3^ where they reported that the sedimentation of nanoparticles could be responsible for the deterioration of nanofluid heat transfer as concentration increases in the enclosure. As a developmental basis, the existing drift flux solver “driftFluxFoam” in OpenFOAM 6 is used to capture the described mixture modeling concept. In addition to the variable thermo-physical properties, only the energy equation is added to the existing drift flux solver to capture the thermal characteristics of the nanofluid. In this solver, the mixture flow is described by the following governing equations of mass, momentum, nanoparticle volume fraction, and energy in Eqs. (5), (6), (7), and (8), respectively.

5 $$\frac{\partial {\rho }_{m}}{\partial t}+\nabla \cdot \left({\rho }_{m}{u}_{m}\right)=0$$

6 $$\frac{\partial \left({\rho }_{m}{u}_{m}\right)}{\partial t}+\nabla \cdot \left({\rho }_{m}{u}_{m}{u}_{m}\right)=-\nabla P+\nabla \cdot \left({\mu }_{m}\nabla {u}_{m}\right)+\nabla \cdot \left(\begin{array}{c}\varphi {\rho }_{p}{u}_{pm}{u}_{pm}+\\ \left(1-\varphi \right){\rho }_{bf}{u}_{bfm}{u}_{bfm}\end{array}\right)+{\rho }_{m}{g}_{k}$$

7 $$\frac{\partial \left({\rho }_{p}\varphi \right)}{\partial t}+\nabla \cdot \left(\varphi {\rho }_{p}{u}_{m}\right)=-\nabla \cdot \left(\varphi {\rho }_{p}{u}_{pm}\right)$$

8 $$\frac{\partial \left({\rho }_{m}{T}_{m}\right)}{\partial t}+\nabla \cdot \left({\rho }_{m}{u}_{m}{T}_{m}\right)=\nabla \cdot \left(\frac{{k}_{m}}{{C}_{pm}}\nabla {T}_{m}\right)$$

where ${u}_{m}$, ${u}_{pm}$, ${u}_{bfm}$, ${\rho }_{p}$, ${\rho }_{bf}$, ${T}_{m}$ represent the mixture velocity vector, the relative velocity vector of nanoparticles, the relative velocity vector of the base fluid, the density of nanoparticles, the density of the base fluid, and the mixture temperature, respectively. The previous definitions of other symbols still hold. As stated previously, the sedimentation or settling of nanoparticles causes the slip motion of nanoparticles. Therefore, the relative velocity of nanoparticles and base fluid resulting from the settling of nanoparticles is assumed to follow the Vesilind^37^ sedimentation model given by Eqs. (9) and (10). A schematic sketch representing the migration of nanoparticles in the gravity direction to settle at the bottom of the enclosure is illustrated in Fig. 1.

9 $${u}_{pm}=\frac{{\rho }_{bf}}{{\rho }_{m}}{u}_{s}{10}^{-A\varphi }$$

10 $${u}_{bfm}=\frac{\varphi {\rho }_{p}}{\left(1-\varphi \right){\rho }_{f}}{u}_{pm}$$

where ${u}_{s}$ and $A$ represent the reference settling velocity vector and settling coefficient and can be determined from experimental data. In absence of dedicated experimental data, the reference nanoparticle settling velocity is calculated from the balance of the buoyancy and viscous force as shown in Eq. (11).

11 $${u}_{s}=\frac{{d}_{p}^{2}\left({\rho }_{p}-{\rho }_{bf}\right)}{18{\mu }_{bf}}g$$

### Two-component model governing equations

**Figure 2:**
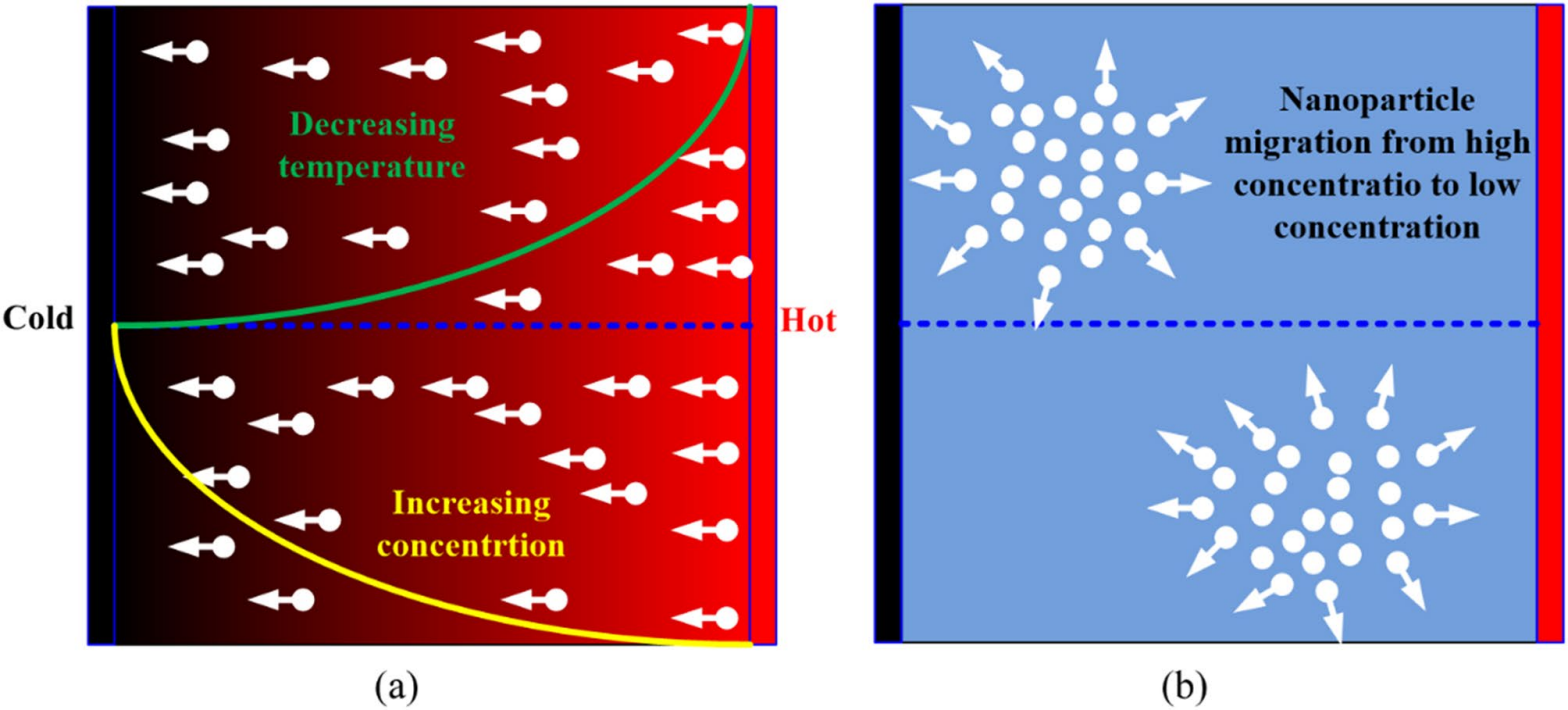
Illustration of nanoparticle slip motions: **(a)** thermophoresis; (b) Brownian diffusions.

The two-component model is a variant of the two-phase modeling approach that was proposed by Buongiorno^18^. This model is predicated on the assumption that nanoparticle slip velocities resulting from various slip mechanisms are responsible for the convective heat transfer enhancement in the nanofluid. The two dominant slip mechanisms, identified by Buongiorno^18^ are; the Brownian diffusion and the thermophoresis diffusion. The random motion of nanoparticles dispersed in a base fluid is termed Brownian diffusion resulting from the continuous collision between nanoparticles and the base fluid molecules while thermophoresis refers to the migration of nanoparticles under the influence of temperature gradient. Schematic sketches illustrating the thermophoresis and the Brownian diffusions of nanoparticles are shown in Fig. 2a,b, respectively. A detailed description of these two slip mechanisms can be found in the textbook of Michaelides^38^ and the review paper of Kleinstreuer and Xu^39^.

Accounting for these two dominant slip mechanisms, a two-component mixture model (base fluid and nanoparticles) has been formulated by Buongiorno as shown in Eqs. (12–15) for mass, momentum, energy, and nanoparticle volume fraction conservation equations, respectively. Starting from the buoyancy-driven convection flow model “buoyantBousinessqPimpleFoam” as a developmental basis, a new solver is developed in this work to implement the Buongiorno model.

12 $$\frac{\partial {\rho }_{m}}{\partial t}+\nabla \cdot \left({\rho }_{m}{u}_{m}\right)=0$$

13 $$\frac{\partial \left({\rho }_{m}{u}_{m}\right)}{\partial t}+\nabla \cdot \left({\rho }_{m}{u}_{m}{u}_{m}\right)=-\nabla P+\nabla \cdot {\mu }_{m}\nabla {u}_{m}+{\rho }_{m}{g}_{k}$$

14 $$\frac{\partial \left({\rho }_{m}{C}_{pm}{T}_{m}\right)}{\partial t}+\nabla \cdot \left({\rho }_{m}{u}_{m}{C}_{pm}{T}_{m}\right)=\nabla \cdot \left({k}_{m}\nabla T\right)-{C}_{pp}{J}_{p}\cdot \nabla {T}_{m}$$

15 $$\frac{\partial \left(\varphi \right)}{\partial t}+{u}_{m}\cdot \nabla \left(\varphi \right)=\frac{1}{{\rho }_{p}}\nabla \cdot {J}_{p}$$

where ${J}_{p}$ and ${C}_{pp}$ represent total nanoparticle mass flux and nanoparticle specific heat capacity, respectively. The total nanoparticle mass flux accounting for the two slip mechanisms, of Brownian diffusion and thermophoresis, is defined by Eq. (16) below.

16 $${J}_{p}={\rho }_{p}\left({D}_{B}\nabla \varphi +{D}_{T}\frac{\nabla {T}_{m}}{{T}_{m}}\right)={\rho }_{p}\left({u}_{B}+\varphi {u}_{T}\right)$$

where ${D}_{B}=\frac{{k}_{B}{T}_{m}}{3\pi {\mu }_{bf}{d}_{p}}$ and ${D}_{T}=\beta \frac{{\mu }_{m}}{{\rho }_{m}}\varphi$ are the Brownian diffusion coefficient and thermophoretic diffusion coefficient, respectively. Therefore, the slip velocity due to Brownian diffusion is obtained as ${u}_{B}=-{D}_{B}\nabla \varphi$ while the slip velocity due to the thermophoretic diffusion is obtained as ${u}_{T}=-{S}_{T}\frac{{\mu }_{m}}{{\rho }_{m}}\frac{\nabla {T}_{m}}{{T}_{m}}$. Additionally, the thermophoretic coefficient (${S}_{T}$) is determined using the expression given by McNab and Meisen^21^ shown in Eq. (17). It is worth mentioning here that the McNab and Meisen expression is independent of the particle size and was originally developed using data of large size particles. Thus, it cannot be scaled down to nanoscale in its present form and might be inappropriate for the prediction of thermophoretic coefficient in a nanofluid.

17 $${S}_{T}=0.26\frac{{k}_{m}}{2{k}_{m}+{k}_{p}}$$

here ${k}_{p}$ is the nanoparticle thermal conductivity. To address the inappropriateness of the McNab and Meisen^21^ model for nanofluid, a correlation was derived by Corcione et al*.*^20^ (shown in Eq. (18)) that described the generated dataset of ${S}_{T}$ which minimized deviation of the errors between simulation data and experimental data of the nanofluid heat transfer. Although the correlation proposed by Corcione et al*.*^20^ did not account for the effect of the Rayleigh number, it is tested in this study to assess its accuracy in the prediction of the nanoparticle thermophoretic diffusion.

18 $${S}_{T}=\left[1.5\times {10}^{4}{\left(\frac{{k}_{p}}{{k}_{bf}}\right)}^{-3}+0.9\right]\cdot \left[-16{\varphi }^{2.35}+0.0195\right]$$

The respective values of the predicted thermophoretic coefficients for nanoparticle concentrations of 1% and 3% are 0.0052 and 0.0055 using Eq. (17) and 0.1986 and 0.1946 using Eq. (18). Unfortunately, none of these values is strong enough to significantly influence the nanofluid heat transfer as it will be shown further down. However, Eq. (17) was developed more mechanistically for large particles. Therefore, it would be more logical to extend this thermophoretic coefficient equation (Eq. (17)) to nanoparticle scale.

## Geometric configuration, boundary conditions, and numerical solution scheme

**Figure 3:**
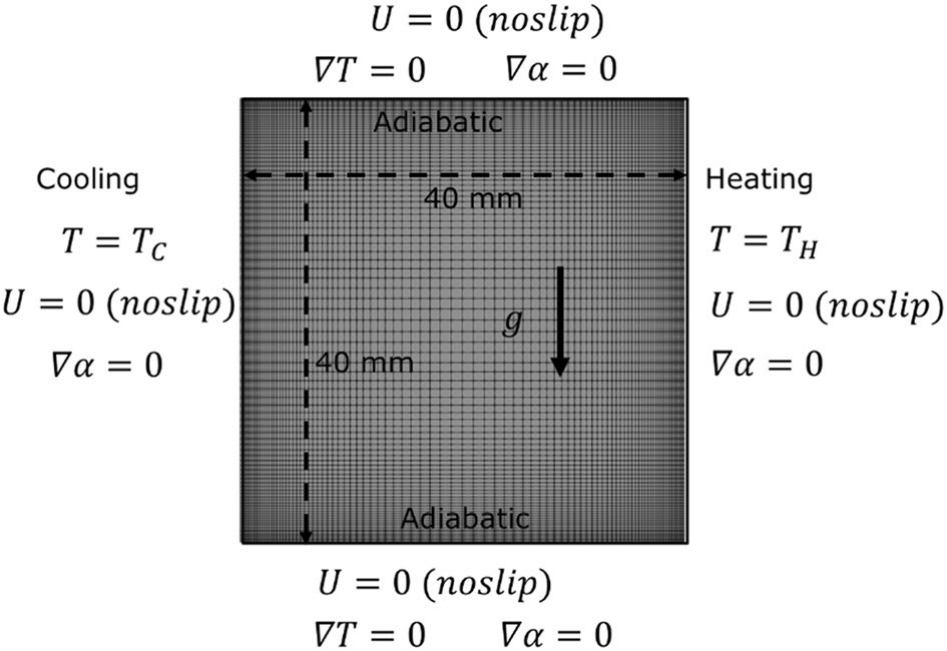
Geometric configuration and boundary conditions.

In this study, a two-dimensional (2D) differentially heated enclosure representing the experimental configuration of Ho et al*.*^4^ is considered. The quadrate enclosure has equal width and height of 40 mm. A constant temperature, ${T}_{H}$ and ${T}_{C}$ boundary conditions are imposed on the right hot side, and left cold side, respectively. Additionally, adiabatic boundary conditions are imposed on the top and bottom sides while non-slip velocity boundary condition is imposed on all four sides. For calculation involving the volume fraction equation, zero particle flux boundary conditions (Neumann boundary condition) in place of constant value boundary conditions (Dirichlet boundary condition) are imposed on all four sides. These boundary conditions are depicted in Fig. 3. As can be seen in Fig. 3, a non-uniform grid is generated with fine mesh near the wall to capture the near-wall behavior of nanofluid and relatively coarse mesh in the central region, where the velocity and temperature gradients are minimal. The nanofluid is made of alumina nanoparticles (Al_2_O_3_) of average particle size of 33 nm dispersed in ultra-pure water (serving as the base fluid) at different volume fractions ranging from 0.1 to 3%.

**Figure 4:**
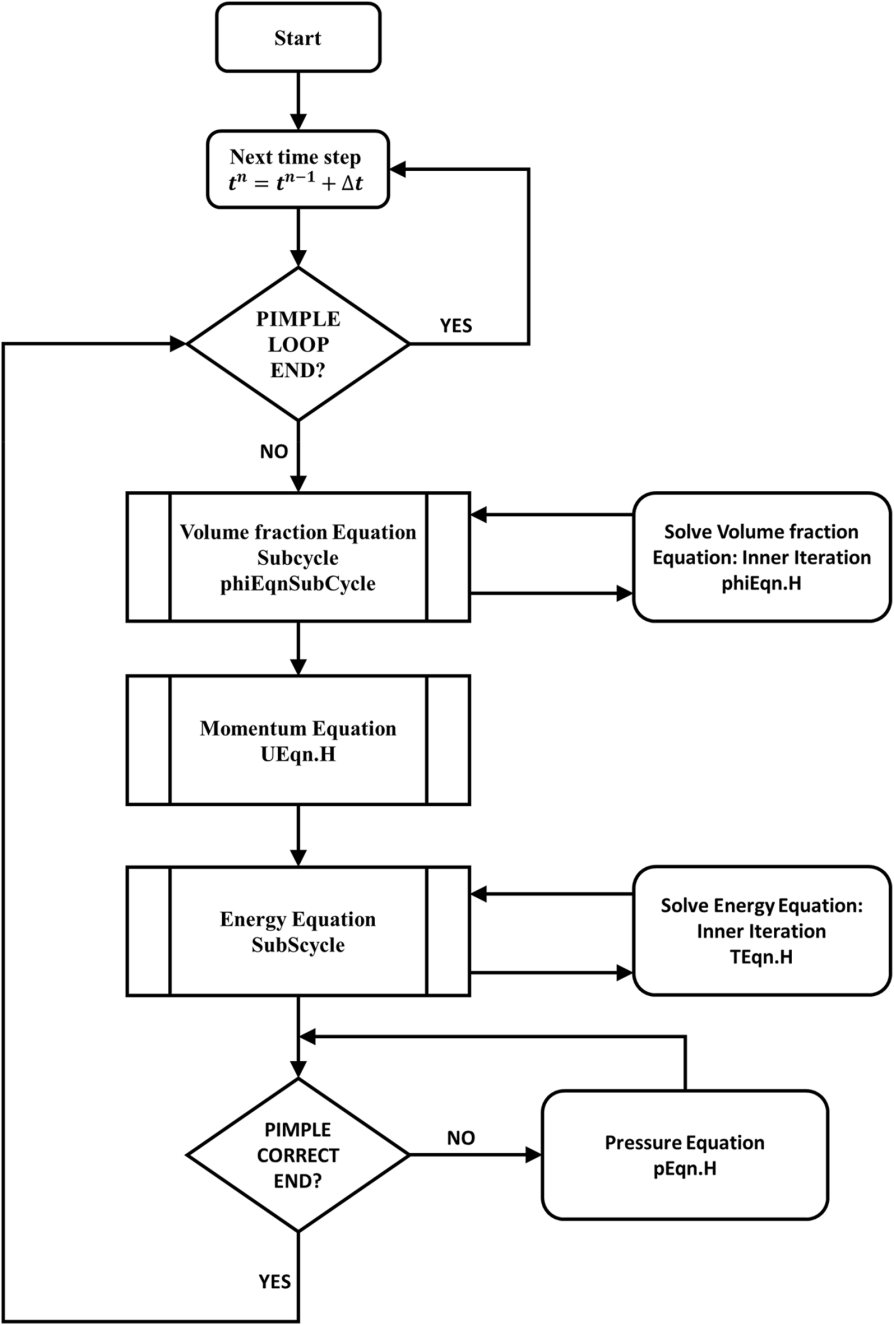
Flow Chart of the OpenFoam PIMPLE algorithm.

The two-dimensional governing equations of the modeling approaches are discretized using the finite volume method. Then a PIMPLE algorithm in OpenFOAM is used, which is a combination of the pressure implicit with the splitting of operator (PISO) algorithm and semi-implicit method for pressure linked equations (SIMPLE) algorithm. More detailed descriptions of these algorithms can be found in the work of Issa^40^ and Ferziger and Peric^41^. The PIMPLE algorithm provides an additional pressure equation (pEqn.H) which is used to decouple the conservation equations (mass, momentum, energy, and volume fraction) such that the resulting system of equations can be solved sequentially. The general flow chart of the PIMPLE algorithm used in this study is shown in Fig. 4. The PIMPLE loop in the algorithm is executed every time step. Within the PIMPLE loop, the sub-cycle for the volume fraction equation (phiEqn.H) is first executed. It should be noted that this section of the loop is only considered for the two-phase models and it is ignored for the single-phase model. Following the volume fraction equation sub-cycle, the momentum equation (UEqn.H) and energy equation (TEqn.H) are executed consecutively before the algorithm enters the pressure correction sub-loop where the pressure equation (pEqn.H) is solved. If the number of the cycle in the PIMPLE loop is restricted to 1, then the algorithm is effectively reduced to a PISO algorithm.

The spatial schemes for gradient, Laplacian, and divergence are Gauss linear, Gauss linear corrected and Gauss linear schemes, respectively. Moreover, the solvers use an adaptive time step which is based on the maximum Courant number (${C}_{o}$) in the domain. The Courant number for the n-degree of freedom is given by Eq. (19). The default value used in this open-source code as a restriction on the Courant number (${C}_{o}\le 0.5$) is set to ensure convergence of the time-marching solver by influencing the maximum time step.

19 $${C}_{o}=\Delta t\sum_{i=1}^{n}\frac{{u}_{i}}{\Delta {x}_{i}}\le 0.5$$

**Figure 5:**
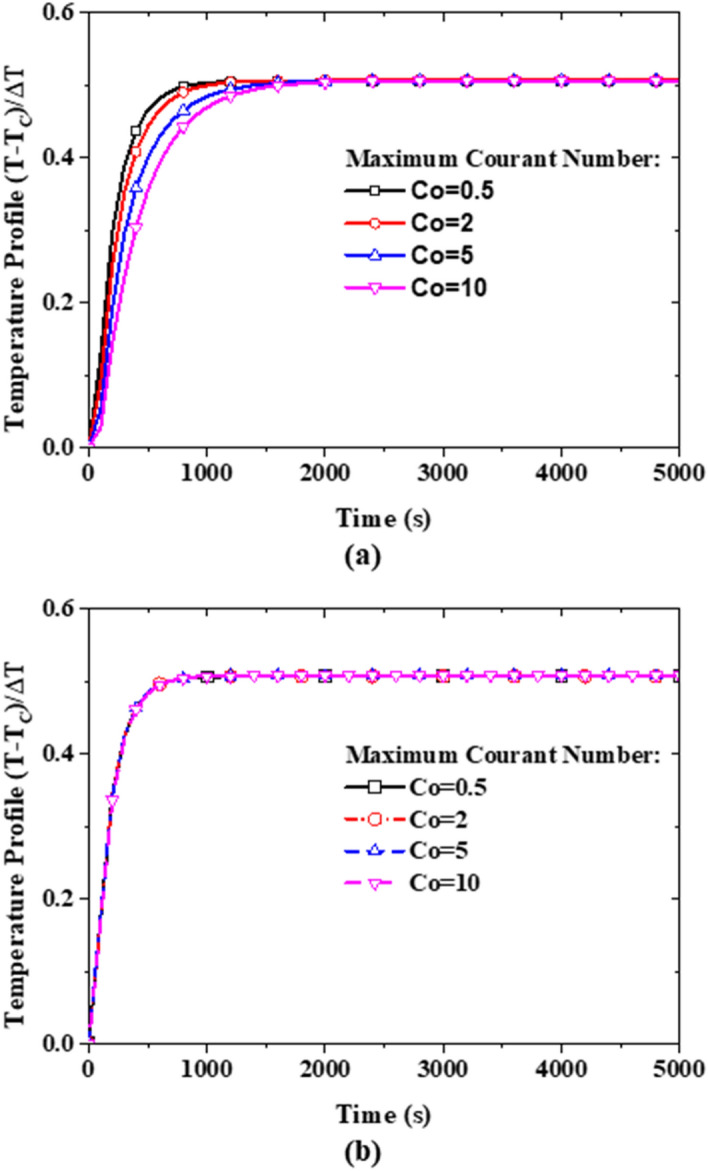
Sensitivity test of the time discretization on the temperature profile at the mid-point of the computational domain using the mixture model: **(a)** with maximum number PIMPLE iterations equal to 3, and **(b)** with a variable number of PIMPLE iterations but with convergence criteria (absolute residual) for the variables set to 10^–6^.

The time step is therefore directly proportional to the mesh spacing (${x}_{i}$). If the spacing is reduced by half, then the time step must be also reduced by half. To assess the solver stability criterion stated above (Eq. (19)), the maximum Courant number was changed to 2, 5, and 10 to ensure that the presently developed solver is sufficiently robust and that the time discretization does not affect the final steady-state results. As it can be seen in Fig. 5a, only the transient part (up to the physical time of ~ 1000 s) of the simulation is affected when increasing the Courant number maximum value limit to 2, 5, and 10. All the Courant numbers produce a converged temperature profile at the mid-point of the computational domain to an asymptotic value. Moreover, the disparity observed using different Courant numbers results from setting a maximum number for the PIMPLE iterations equal to 3. This sensitivity to time-step is eliminated by setting instead a convergence criterion of 10^–6^ for each variable. As shown in Fig. 5b, irrespective of the maximum Courant number, the temperature profiles at the mid-point of the domain converge throughout the simulation physical time. This way, the PIMPLE iteration continues until these convergence criteria are achieved. The time-step discretization sensitivity shown in Fig. 5 is for the mixture model. However, this behavior is also true for both the single-phase model and two-component model.

Based on the above findings, a maximum Courant Number is kept to 0.5 (as this had no dramatic impact on the simulation time) and the absolute residuals of velocity, temperature, pressure, and nanoparticle volume fraction are restricted to below 10^–6^ as a convergence criterion during the iteration process in the present study. At the attainment of the asymptotic solution, the heat flux at the hot boundary surface is equal to the one at the cold boundary surface which is calculated using Eq. (20).

20 $${q}^{"}=-{k}_{m}\cdot \frac{\partial T}{\partial n}$$

where $n$ denotes the normal to the surface. Subsequently, the Rayleigh number, the heat transfer coefficient, and the Nusselt number are computed using Eqs. (21), (22), and (23), respectively.

21 $$Ra=\frac{g{\rho }_{m}^{2}{\beta }_{m}\Delta T{H}^{3}}{{k}_{m}{\mu }_{m}}$$

22 $$h=\frac{{\overline{q} }^{"}}{\Delta T}$$

23 $$Nu=\frac{{\overline{q} }^{"}H}{\Delta T{k}_{m}}$$

where ${\overline{q} }^{"}$ is the spatial average of the heat flux on the hot boundary surface (which is the same for the cold boundary surface), $\Delta T$ is the temperature difference between the cold surface (${T}_{C}$) and hot surface (${T}_{H}$).

Using the single-phase and the two variants of the two-phase model described earlier in “Mathematical model”, a grid independence test is performed on six grids 60 × 60, 80 × 80, 100 × 100, 120 × 120, 130 × 130, and 140 × 140 as shown in Table 3. The predicted average Nusselt number at the hot side for Ra = 6 × 10^6^ (the maximum Rayleigh number considered in this study) at a nanoparticles volume fraction of 3% shows that variation with grid becomes infinitesimal after the grid size of 100 × 100 as there is less than 0.1% difference in the Nusselt number between the 100 × 100 and 120 × 120 grid size. Therefore, subsequent computations are performed using 100 × 100 grid size. Table 3 further shows a grid-independence study for the three models.

**Table 3:** Grid independent test results for Ra = 6 × 10^6^ and 3% nanoparticle concentration.

	Predicted average Nusselt number		
Grid size	Single-phase model	Two-component model	Mixture model
60 × 60	13.554	13.482	11.926
80 × 80	13.511	13.458	11.907
100 × 100	13.488	13.446	11.899
120 × 120	13.474	13.439	11.894
130 × 130	13.469	13.437	11.893
140 × 140	13.465	13.435	11.891

## Validation of the models and discussion of results

**Figure 6:**
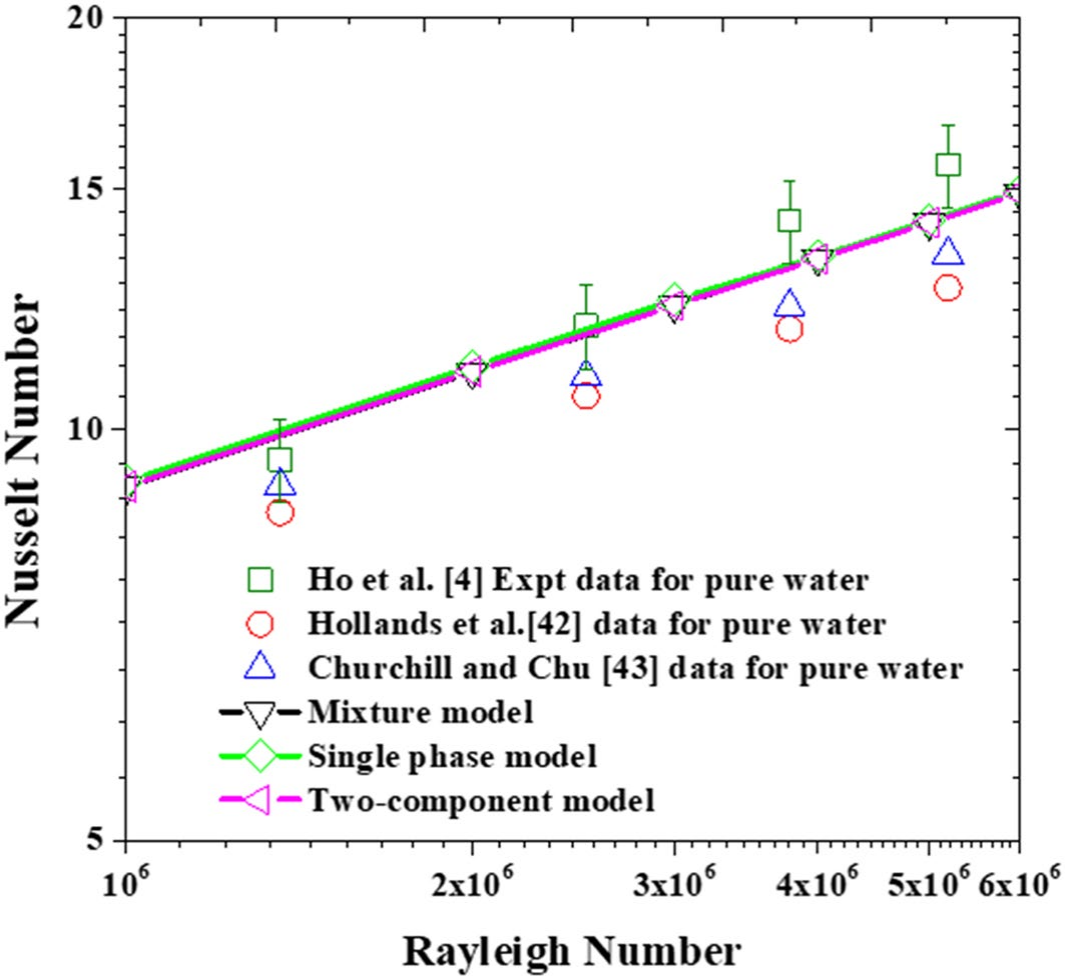
Models’ validation with Ho et al*.*^4^ experimental data for pure water.

In this section, the three modeling approaches are first validated against the experimental data of Ho et al*.*^4^ for the ultra-pure water cases. Hence, simulations have been conducted for laminar natural convection in the square cavity (see Fig. 3). This elementary yet compulsory step is required to evaluate the accuracy and reliability of the models’ implementation in the open-source code. As can be seen in Fig. 6, all three modeling approaches are able to reproduce the experimentally obtained average Nusselt numbers, reported by Ho et al*.*, within the uncertainty range. Furthermore, as to be expected, the predictions from the three approaches converge to a single curve since the working fluid does not contain nanoparticles. That is, in the absence of suspended nanoparticles, the solution reverse back to the pure water predictions. This implies that all terms containing the void fraction variable (*φ*), including the ones in the thermophysical properties correlations, are correctly eliminated for the solver to reduce to its basic form; valid for pure water. Added in the figure are the experimental data of Holland et al*.*^42^, and Churchill and Chu^43^ which are represented by the correlations given in Eqs. (24) and (25), respectively. At first sight, the discrepancy observed between the values obtained using these two correlations and the experimental measurements of Ho et al*.* might be of concern. However, by recognizing the fact that these correlations were generated from many data, spanning over a wide range of Rayleigh numbers, and extending from the laminar to the turbulent flow regimes then, such differences can be justified.

24 $$Nu=1+1.44\left(1-\frac{1708}{Ra}\right)+\left[{\left(\frac{Ra}{5830}\right)}^{1/3}-1\right]+2{\left[\frac{{Ra}^{1/3}}{140}\right]}^{\left[1-\mathrm{ln}\left({Ra}^{1/3}/140\right)\right]}$$

25 $$Nu=0.289{Ra}^{1/4}$$

**Figure 7:**
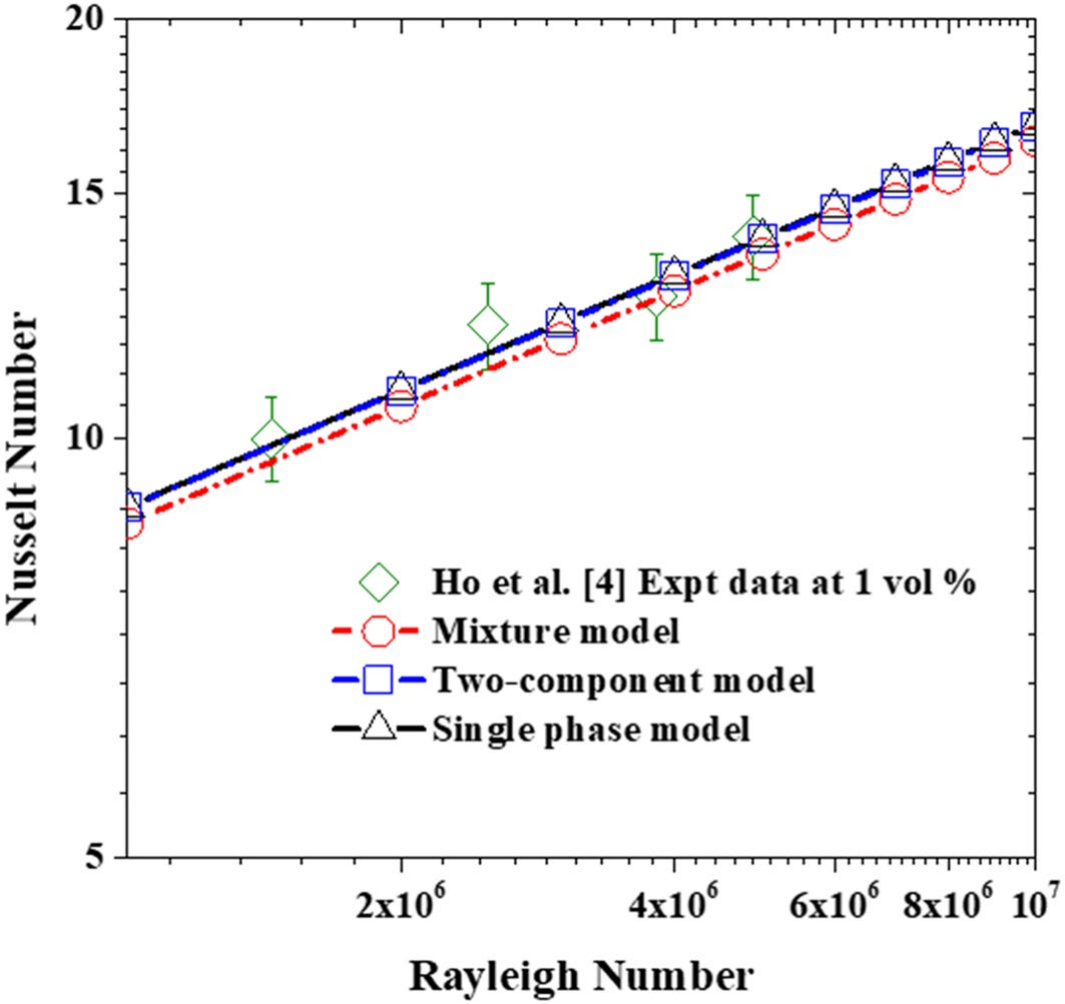
Comparison of the simulation data with the experimental data using 1% nanoparticle concentration.

Also, for the cases with nanoparticles concentration of 1%, all three models can reasonably predict the experimental measurements within the ± 7% error band (see Fig. 7). It must be stated that the Brownian diffusion and Thermophoretic diffusion (computed using the correlation proposed by Corcione et al*.*^20^ for the thermophoretic parameter) has no significant impact on the nanofluid heat transfer rate, as the resulting deviation of the two-component model from the single-phase model is no more than ~ 0.2%. A second interesting deduction to be made from these plots is that there is no apparent added value by using the more complex two-component model instead of the basic one for such a low concentration. As for the third nanoparticle slip mechanism (sedimentation), the deviation of the mixture model from the single-phase model is ~ 3%. Although this model is also able to predict the experimental data within the uncertainty limit as can be seen in Fig. 7, the model returns somewhat underpredicted values for the Nusselt number in comparison to the other two models and the experimental data. This is a twofold observation, firstly accounting for sedimentation does result in heat transfer impairment and secondly, these underpredicted values for the Nusselt number give a hint to the fact that the nanoparticle sedimentation term might be requiring some adjustment to better reflect the reference experimental data. These last two points are further investigated and discussed in “Proposed combination of the nanoparticle’s thermophoresis diffusion, Brownian diffusion, and sedimentation in a two-phase model” below.

**Figure 8:**
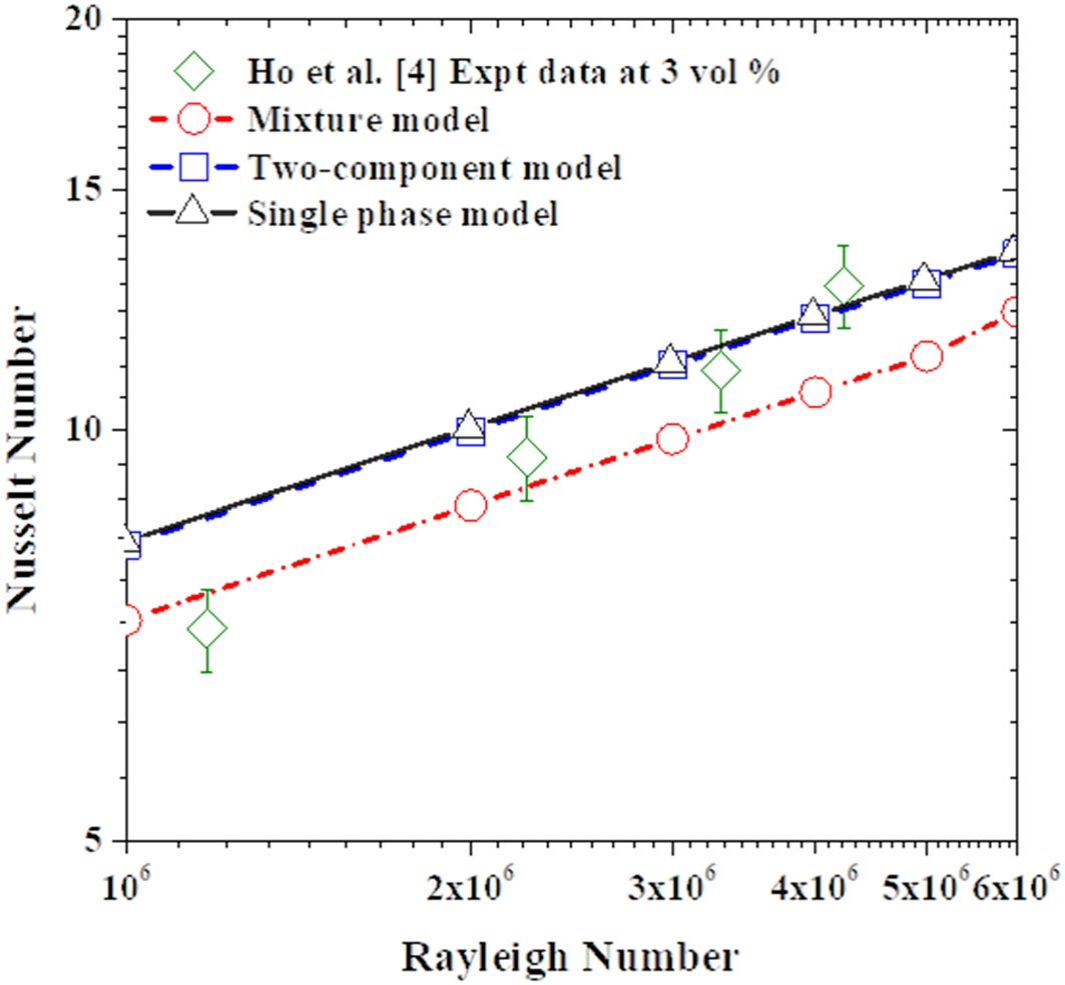
Comparison of the simulation data with the experimental data using 3% nanofluid.

As shown in Fig. 8, once the volume fraction of the nanoparticles is increased to 3%, clear deviations are observed between the predicted data of the single-phase model and the mixture model. Comparing the predictions obtained by these two models with the experimental data, very intriguing observations can be made. Firstly, in the low Rayleigh number range (up to 2.3 × 10^6^ approximately), the mixture model predictions are much closer to the experimental data than the ones obtained with the single-phase model. Then, for the cases with higher Rayleigh numbers, the single-phase model predictions become superior as the mixture model is observed to significantly underpredict the experimental data. This is suggesting that the current sedimentation term, in the mixture model, is providing the right magnitude for the slip mechanism at the low Rayleigh number range, but the same cannot be said for Rayleigh number values larger than 2.3 × 10^6^. Hence, by reference to the Eqs. (6), (9), (10), and (11), the sedimentation term should also account for the Rayleigh number effects in addition to the nanoparticle concentration ones. This finding is in agreement with the common understanding that at slow flow motion (i.e., low Rayleigh number) the nanosuspensions are more prone to sedimentation, but by increasing the flow motion this effect becomes less significant and the nanoparticles get carried away by the flow stream.

**Figure 9:**
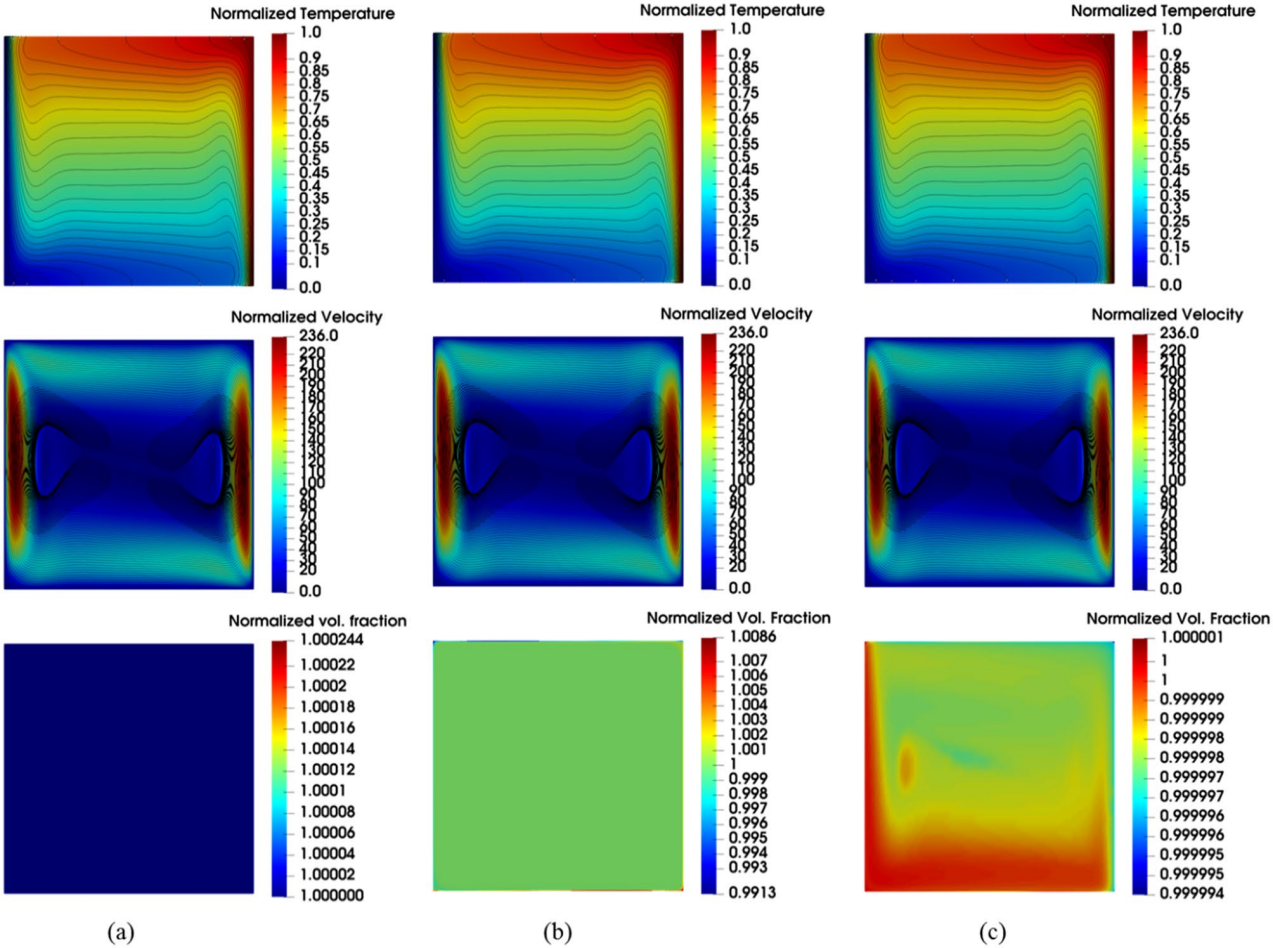
Isotherm (top) calculated as a dimensionless parameter $(T-{T}_{C})/\Delta T$, Streamline (middle) calculated as dimensionless parameter $u\rho {c}_{p}L/k$, and contour of volume fraction of nanoparticle (bottom) calculated as dimensionless parameter $\varphi /{\varphi }_{avg}$ using the three modeling approaches with nanoparticle concentration of 1% at Ra = 1 × 10^6^: **(a)** single-phase model, **(b)** mixture model, and **(c)** two-component model.

Also, by analyzing the two-component model predictions, it seems that the thermophoretic diffusion and Brownian diffusion terms are not strong enough to influence the heat transfer rate. Actually, the differences in the predictions by the two-component model from the ones by the single-phase model are hardly visible. Here again, the upgrade of the modeling approach, by accounting for these two slip mechanisms and solving an additional equation for the nanoparticles volume faction, does not seem to provide any apparent benefits. However, the same cannot be said for the field distribution of the nanoparticle volume fractions illustrated in Fig. 9. In this figure is not easy to distinguish between the velocity and temperature fields predicted by the three models but the difference in the nanoparticle volume fraction is obvious. As can be seen in Fig. 9c, both the two-component model and mixture model are able to capture the nanoparticle non-uniformity in the concentration. The apparent drift of the nanoparticles from the hot to the cold regions in the two-component model results is due to the inclusion of the thermophoretic diffusion term in this model, while the impact of the Brownian diffusion term is less noticeable. As for the other two models; the nanoparticles are uniformly distributed in a single-phase model (see Fig. 9a) conforming with its basic assumption, while the nanoparticles sedimentation is captured by the mixture model as can be seen in Fig. 9b. As intended, the mixture model is predicting the nanoparticles settling at the bottom of the enclosure due to the sedimentation term effects. It is interesting to note that these high/low nanoparticle concentrations are observed to be taking place locally at the vicinity of the top/bottom isothermal walls, yet the resulting effect is propagated to vertical hot and cold walls. Furthermore, by comparing the temperature and velocity fields in Fig. 9a,b, these effects do not seem to have a significant impact on the core of the natural convection rotational motion.

**Figure 10:**
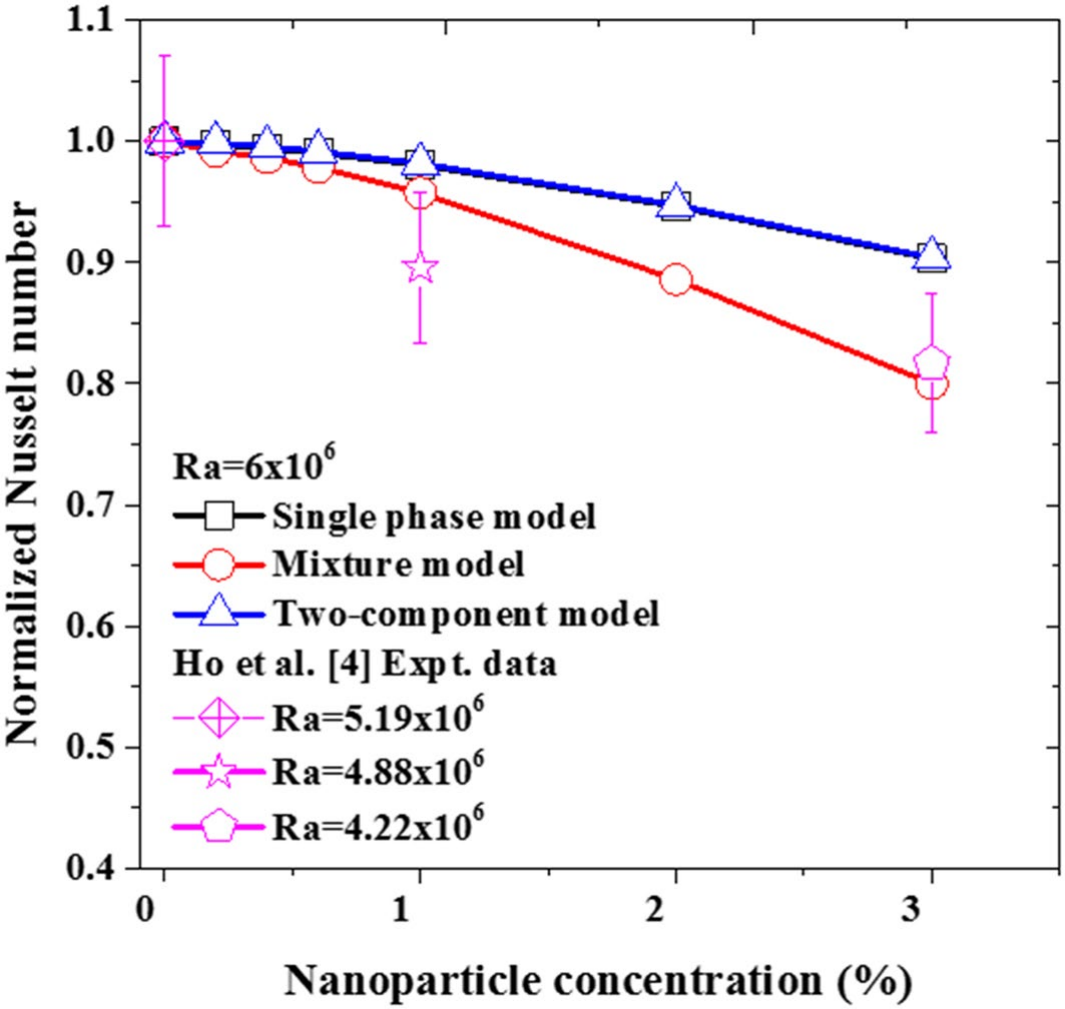
The heat transfer characteristics of the nanofluid with increasing nanoparticle concentration at Ra = 6 × 10^6^.

To closely assess the three modeling approaches’ ability to predict the heat transfer impairment due to the nanoparticles increased concentration in the nanofluid, the predictions of the models for the Nusselt number (normalized by the corresponding pure water case) as a function of nanoparticle concentration is plotted in Fig. 10. Interestingly, in conformity with the experimental data, the three modeling approaches predict the deterioration of nanofluid heat transfer as the nanoparticle concentration increases. Quantitatively, for the case of $\varphi =3\%$, the average values of the predicted deterioration by the mixture, single-phase, and two-component models are 19.9%, 9.7%, and 9.5%, respectively. Comparing these values with the corresponding deterioration in Ho et al*.*^4^ experimental data which is 18.3%, it can be said that the mixture model captures better the heat transfer deterioration, while the other two models are of equal performance.

In light of the above analysis, the single-phase model is confirmed to be inadequate for a quantitative prediction of nanofluid natural convection heat transfer at high nanoparticles concentration (e.g., at $\varphi =3\%$), especially at low Rayleigh numbers, where sedimentation effects can no longer be neglected. The two-phase mixture model, on the other hand, is over predicting the heat transfer deterioration for nanofluid with high nanoparticles concentration, but only for the high Rayleigh number cases. This model seems to be also missing physical flow phenomena associated with the thermophoretic diffusion and, to some extent, the Brownian motion slip mechanisms. Lastly, the two-component model, on its current version, does not seem to provide significant benefits to justify the additional modeling complexity. From this model results, the dominant slip mechanism is the thermophoretic diffusion, while from the mixture model results the sedimentation slip mechanism seems to be also playing an important role. Hence, to further assess the impact that these two terms (sedimentation and thermophoretic diffusion) might have on the heat transfer rate, it is possible to adjust them through their controlling parameters (settling coefficient (A) and thermophoretic coefficient (${S}_{T}$)) as detailed in “Proposed combination of the nanoparticle’s thermophoresis diffusion, Brownian diffusion, and sedimentation in a two-phase model” below.

## Proposed combination of the nanoparticle’s thermophoresis diffusion, Brownian diffusion, and sedimentation in a two-phase model

**Figure 11:**
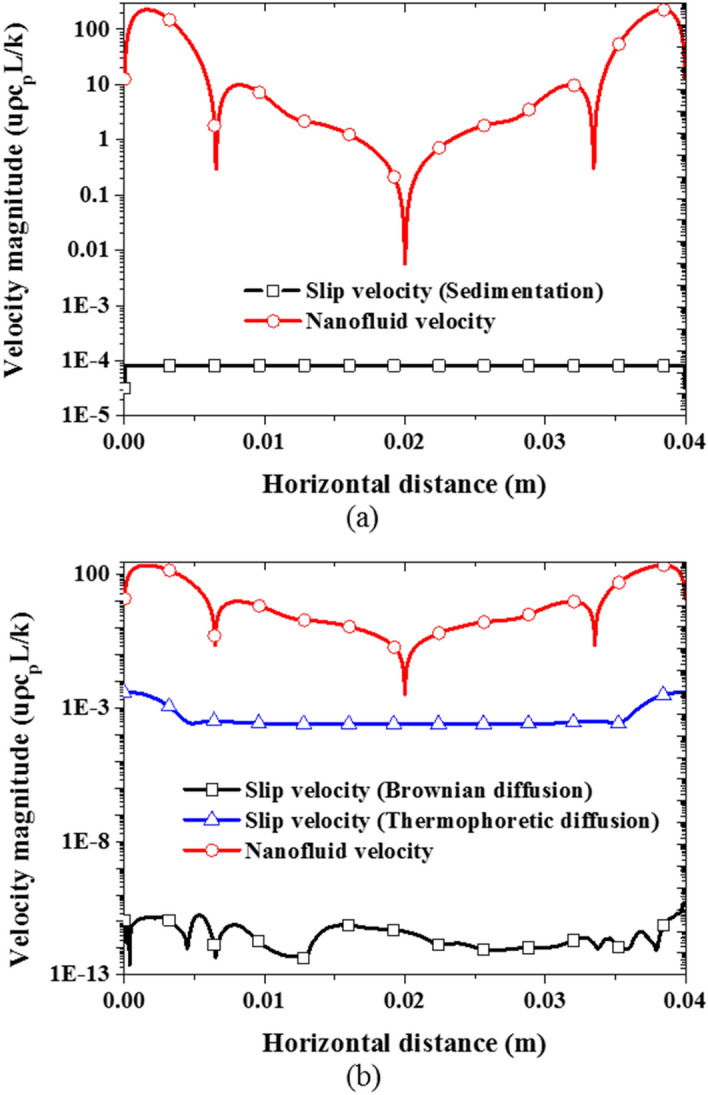
Comparison of the predicted slip velocities with the nanofluid velocity with the nanoparticle volume fraction of 1% at Ra = 1 × 10^6^ at the middle plane: **(a)** mixture model, and **(b)** two-component model.

Observably, in the two-component model (see Fig. 11b), the slip velocity due to the thermophoretic diffusion is comparable (in terms of magnitude) with the slip velocity (shown in Fig. 11a) resulting from nanoparticle sedimentation in the mixture model. In agreement with the observation made above, the slip velocity resulting from the thermophoretic diffusion is far higher than that of Brownian diffusion as can be seen in Fig. 11b. As nanoparticles migrate from the hot region to the cold region due to the thermophoresis diffusion causing non-uniformity in the particle distribution, the Brownian diffusion is supposed to move the nanoparticles in the opposite direction of the particle concentration gradient to create a homogeneous distribution of the nanoparticles. However, by comparing the magnitude of these slip velocities, it appears that the Brownian diffusion does not have any significant contribution to the nanoparticle migration in the nanofluid. Therefore, it can be argued that the two dominant slip mechanisms are the thermophoretic diffusion and sedimentation of nanoparticles. Thus, there is a need for a two-phase formulation able to account for both; the thermophoretic diffusion and sedimentation of nanoparticles. However, as noted above, adjustment for these two terms, through their respective coefficients $A$ and ${S}_{T}$ parameters, is required. Previously, Esfandiary et al*.*^44^, and Pakravan and Yaghoubi^45^ have attempted to account for nanoparticle slip effect due to Brownian and thermophoretic diffusions (without sedimentation) using a different formulation from Buongiorno^18^.

In light of this, a new numerical formulation based on the drift-flux model is proposed here. This model combines the effects of nanoparticle sedimentation, Brownian diffusion, and thermophoretic diffusion in the energy, momentum, and nanoparticle volume fraction equations as can be seen in Eqs. (26)–(29) where the previous definitions of all parameters still hold. In this formulation, the nanoparticle slip velocities represented by ${u}_{pm}$, ${u}_{B}$ and ${u}_{T}$ correspond to sedimentation, Brownian diffusion, and thermophoretic diffusions, respectively. These parameters have been previously defined in “Mathematical model” of this article. Using the OpenFOAM mixture model solver, “driftFluxFoam” as a developmental basis, a new solver is developed to implement the proposed model.

26 $$\frac{\partial {\rho }_{m}}{\partial t}+\nabla \cdot \left({\rho }_{m}{u}_{m}\right)=0$$

27 $$\frac{\partial \left({\rho }_{m}{u}_{m}\right)}{\partial t}+\nabla \cdot \left({\rho }_{m}{u}_{m}{u}_{m}\right)=-\nabla P+\nabla \cdot \left({\mu }_{m}\nabla {u}_{m}\right)+\nabla \cdot \left(\begin{array}{c}\varphi {\rho }_{p}{u}_{pm}{u}_{pm}+\\ \varphi {\rho }_{p}{u}_{T}{u}_{T}+\\ \varphi {\rho }_{p}{u}_{B}{u}_{B}\\ \left(1-\varphi \right){\rho }_{bf}{u}_{bfm}{u}_{bfm}\end{array}\right)+{\rho }_{m}{g}_{k}$$

28 $$\frac{\partial \left({\rho }_{p}\varphi \right)}{\partial t}+\nabla \cdot \left(\varphi {\rho }_{p}{u}_{m}\right)=-\nabla \cdot \left(\varphi {\rho }_{p}{u}_{pm}+\varphi {\rho }_{p}{u}_{T}+\varphi {\rho }_{p}{u}_{B}\right)$$

29 $$\frac{\partial \left({\rho }_{m}{C}_{pm}{T}_{m}\right)}{\partial t}+\nabla \cdot \left({\rho }_{m}{u}_{m}{C}_{pm}{T}_{m}\right)=\nabla \cdot \left({k}_{m}\nabla T\right)-{C}_{pp}{J}_{p}\cdot \nabla {T}_{m}$$

**Figure 12:**
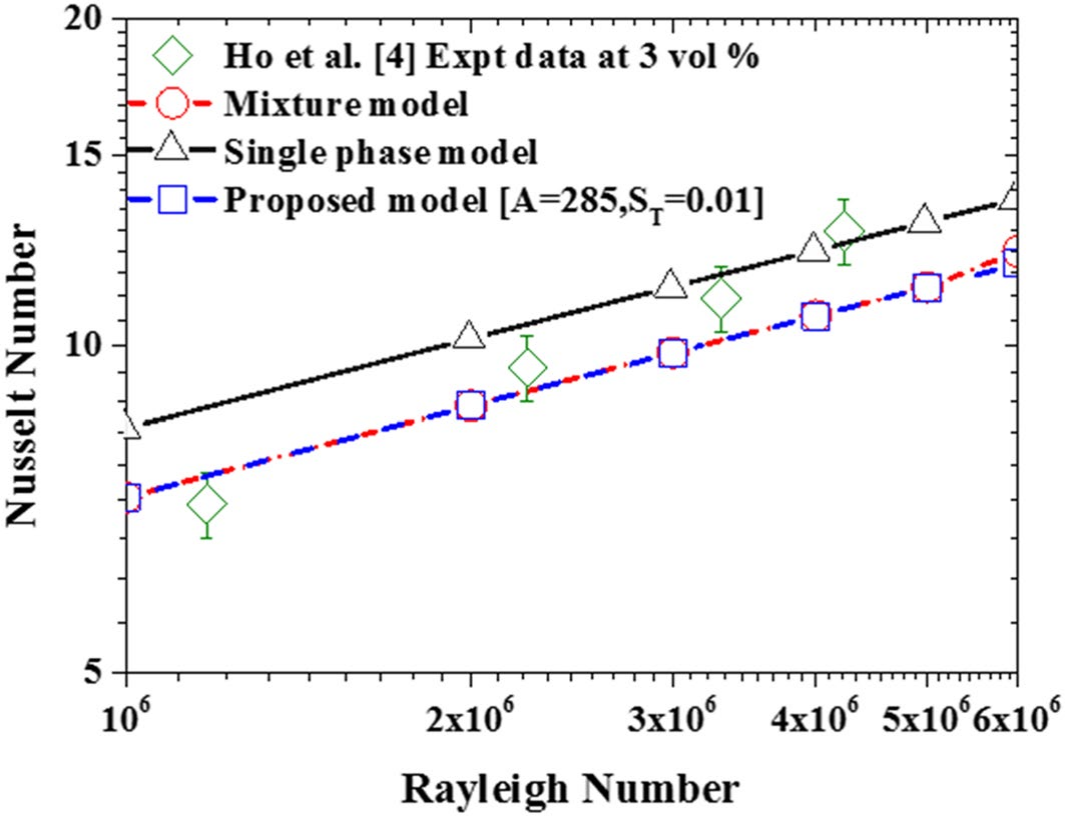
Comparison of the proposed model with single-phase model and mixture model using the default values of settling and thermophoretic coefficients.

Using the default settling coefficient $A=285.84$ and thermophoretic coefficient ${S}_{T}=0.01$ (between the value predicted by Eqs. (17) and (18)) as the baseline parameters, Fig. 12 shows that the prediction by the proposed model is now clearly different from the single-phase model but almost the same as the mixture model since the thermophoretic diffusion is weak at this value of the thermophoretic coefficient.

**Figure 13:**
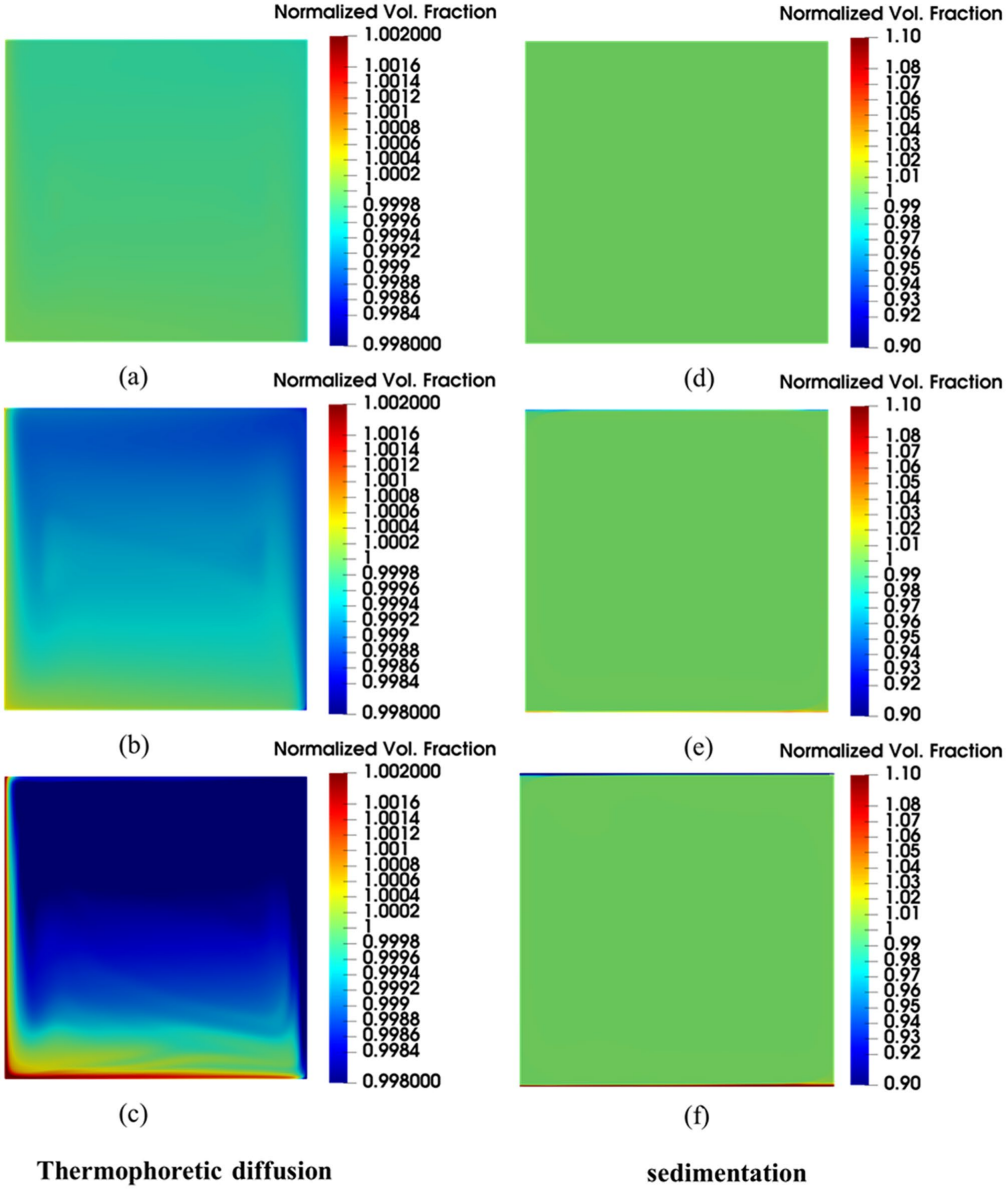
Volume fraction ($\varphi /{\varphi }_{avg}$) distribution of nanoparticle concentration resulting from the slip mechanisms at Ra = 6 × 10^6^ and the average concentration of 3% using: **(a)** ${S}_{T}=0.01$, **(b)** ${S}_{T}=2$, **(c)** ${S}_{T}=5$, **(d)** $A=285$, **(e)** $A=50$, **(f)** $A=20$.

**Figure 14:**
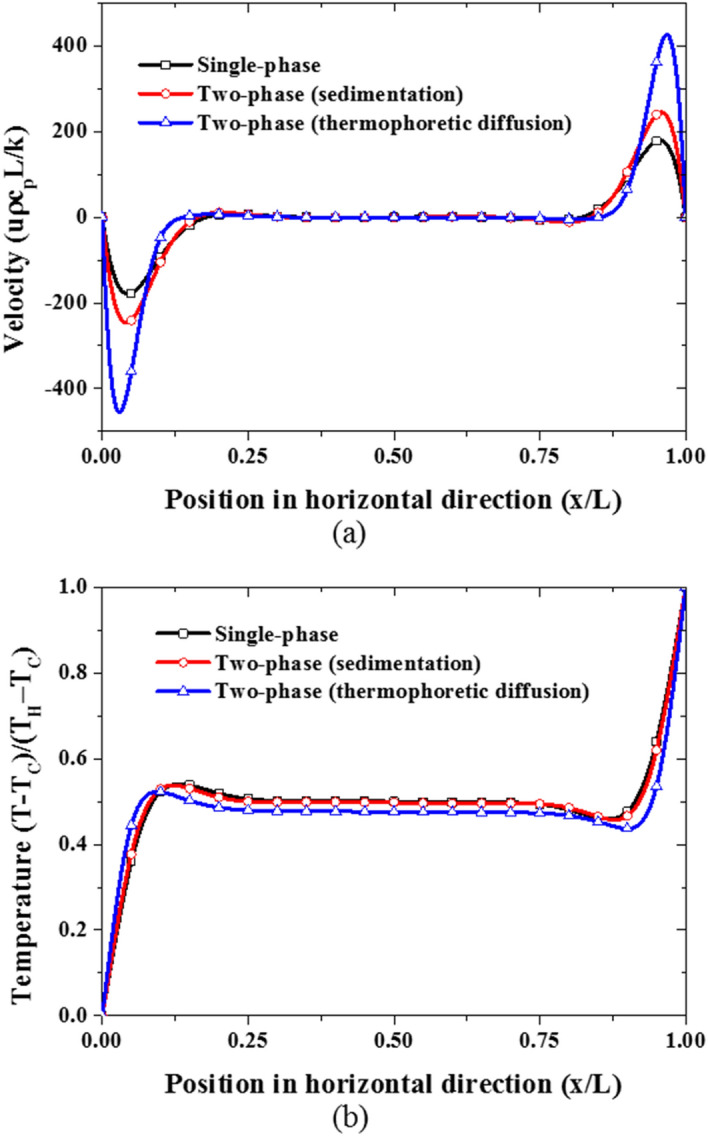
(Red line) Impact of and sedimentation ($A=50$, ${S}_{T}=0.01$), (blue line) thermophoretic diffusion ($A=285.84$, ${S}_{T}=0.5$), **(a)** vertical velocity profile, **(b)** temperature profile at mid-plane of the cavity.

With this solution framework, the two coefficients ($A$ and ${S}_{T}$) can be adjusted to increase the strength of their respective slip mechanisms in nanofluids to investigate their impact on the nanofluid natural convection heat transfer. At first, keeping the settling coefficient constant ($A=285.84$), different computations are performed with a different thermophoretic coefficient (${S}_{T}$) values of 0.01, 2, 5, and the impact of these values on the distribution of the nanoparticle concentration are shown in Fig. 13a,b,c, respectively. These figures show that the nanoparticle becomes more concentrated in the cold regions as the thermophoretic diffusion increases by increasing the thermophoretic coefficient. As a result, the diffusion of nanoparticles from hot regions to cold regions becomes stronger. Secondly, keeping the thermophoretic coefficient constant (${S}_{T}=0.01$), different computations are performed using different values of the settling coefficient ($A$): 285, 50, 20, and their effects on the distribution of the nanoparticle concentration are shown in Fig. 13d,e,f, respectively. As the settling coefficient increases, more nanoparticles are settled at the bottom of the enclosure while creating a thin layer of purer fluid at the top. Additionally, the nanofluid velocity profile and temperature profile are also affected by these slip mechanisms as can be seen in Fig. 14a,b, respectively. Perhaps the most important observation to note here is that the proposed model has a demonstrative capability to capture both; the thermophoretic diffusion and the sedimentation of nanoparticles at the same time, hence, giving a more complete description of the flow physics in comparison to all the other models examined above.

**Figure 15:**
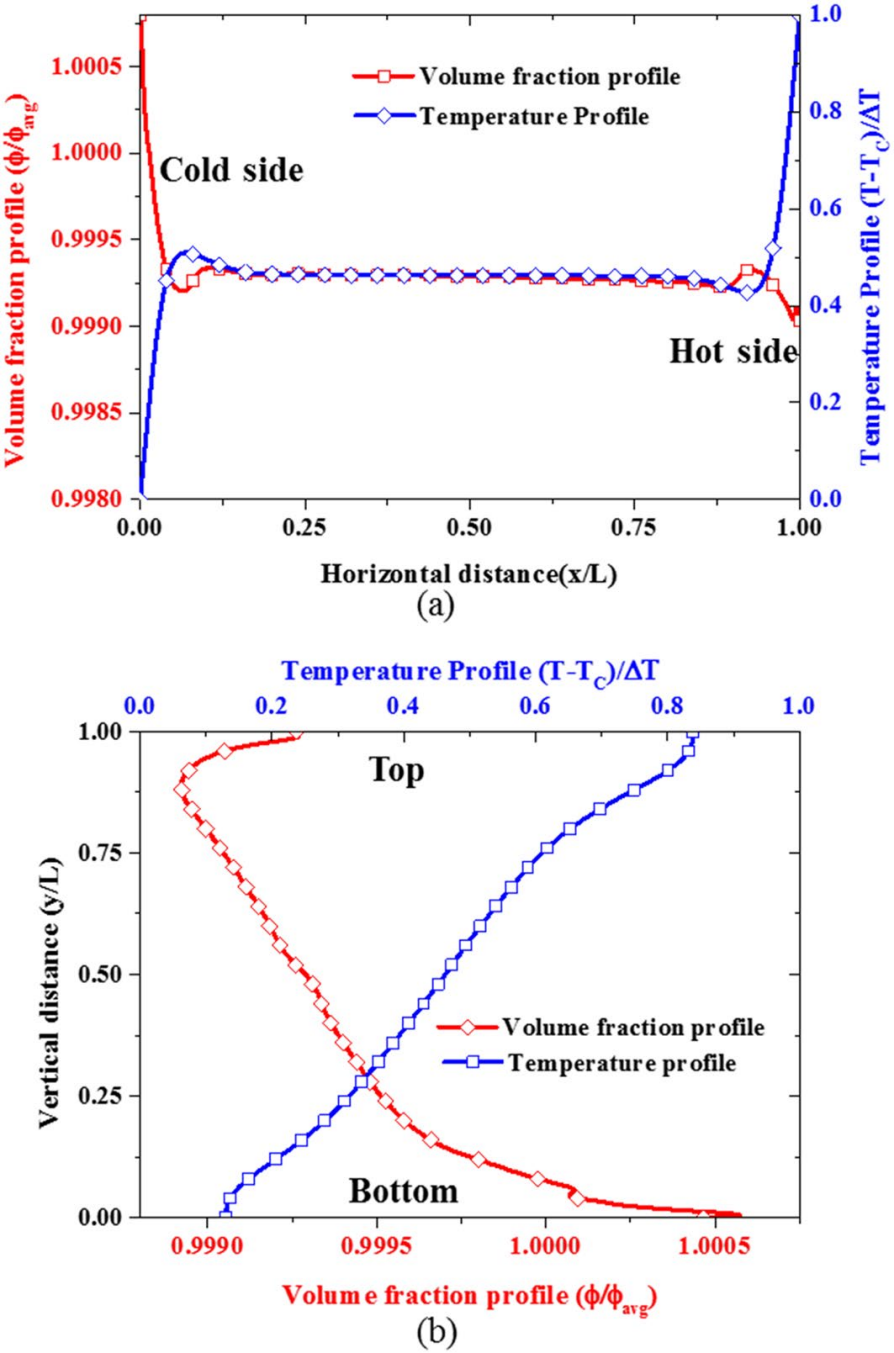
Variation of the temperature and volume fraction profiles at Ra = 6 × 10^6^, nanoparticle concentration of 3%, A = 150.0 and ${S}_{T}=0.1$: **(a)** horizontal mid-plane, and **(b)** vertical mid-plane.

Figure 15 shows the nanoparticle volume fraction distribution and temperature distribution along horizontal and vertical mid-plane. These results indicate that nanoparticle volume fraction is lesser at the hot wall where the temperature is higher due to the thermophoretic diffusion of nanoparticles from the hot wall to the cold wall as shown in Fig. 15a. Likewise, since the top region is hotter than the bottom region as can be seen in Fig. 15b, thermophoretic diffusion also contributes to the downward migration of the nanoparticles from the top to the bottom of the cavity which subsequently results in nanoparticle deposition on the bottom wall. Coincidentally, the gravity acts in the downward direction as well, thereby causing nanoparticle sedimentation on the bottom in addition to the thermophoretic diffusion of nanoparticles. This implies that the migration of nanoparticles from the hot wall to the cold wall through thermophoretic diffusion favor and enhance heat transfer across the enclosure while the deposition of nanoparticles at the bottom wall causes the formation of a stagnant thin layer at the top and bottom walls which could lead to the deterioration of the nanofluid heat transfer.

**Figure 16:**
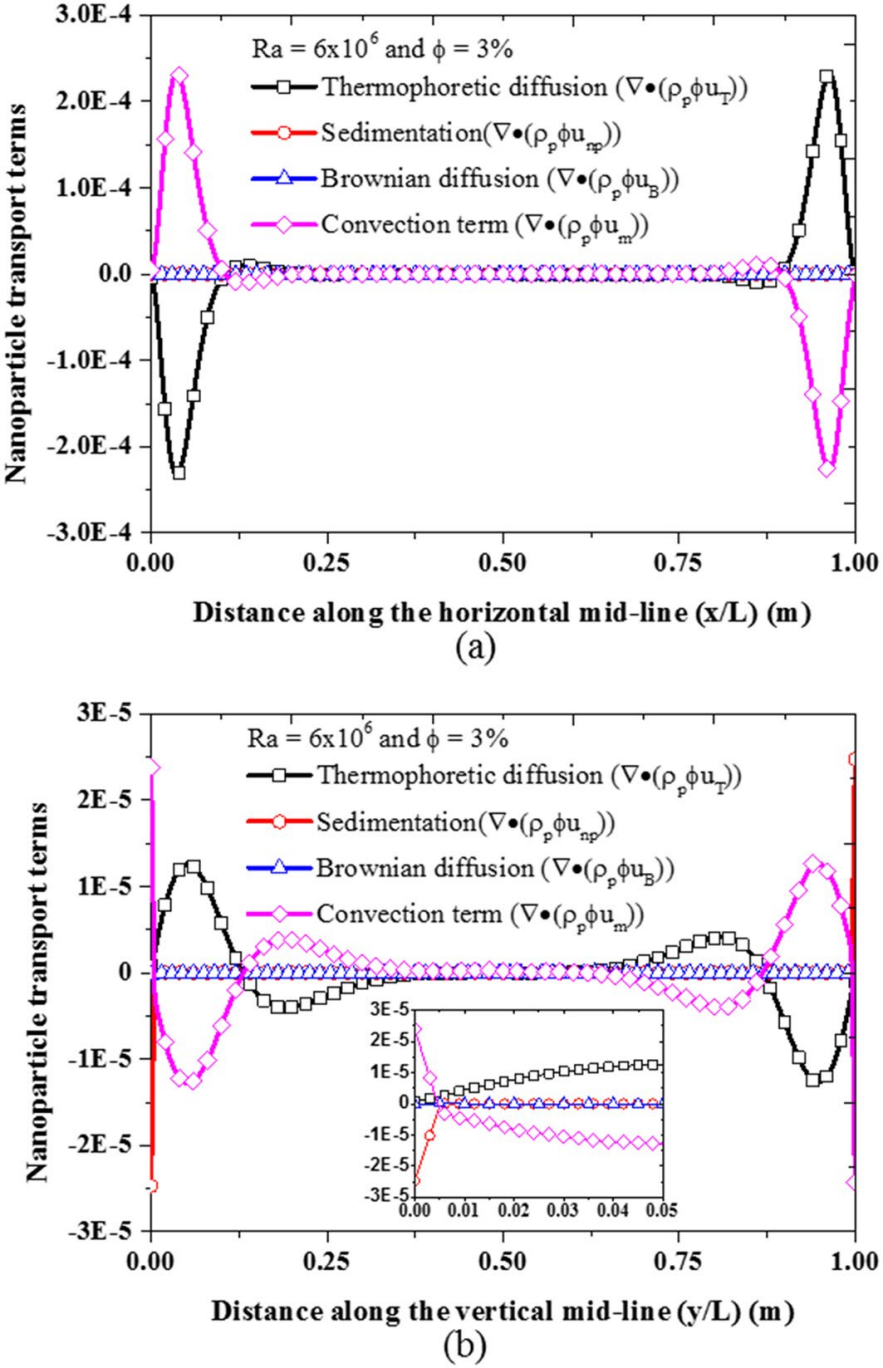
Migration of nanoparticles due to the three slip mechanisms at A = 150.0 and ${S}_{T}=0.1$: **(a)** horizontal mid-plane and **(b)** vertical mid-plane.

To gain further insight into the nanoparticle transport in nanofluid flow, the terms in the nanoparticle volume fraction equation corresponding to the contribution of the three nanoparticle transport phenomena are examined as shown in Fig. 16. As earlier stated, the contribution of the Brownian diffusion to the migration of the nanoparticle is relatively insignificant as can be seen in Fig. 16. Moreover, the thermophoretic diffusion is stronger in the horizontal direction near the hot and cold walls than near the top and bottom walls as shown in Fig. 16a,b. This is because the temperature gradients near the hot and cold walls are steeper than the temperature gradient near the top and bottom walls (see Fig. 15). Additionally, only thermophoretic diffusion contributes to the horizontal migration of nanoparticles as shown in Fig. 16a whereas both thermophoretic diffusion and sedimentation by gravity are responsible for the movement of nanoparticles to the bottom and top of the cavity as shown by the inset in Fig. 16b.

**Figure 17:**
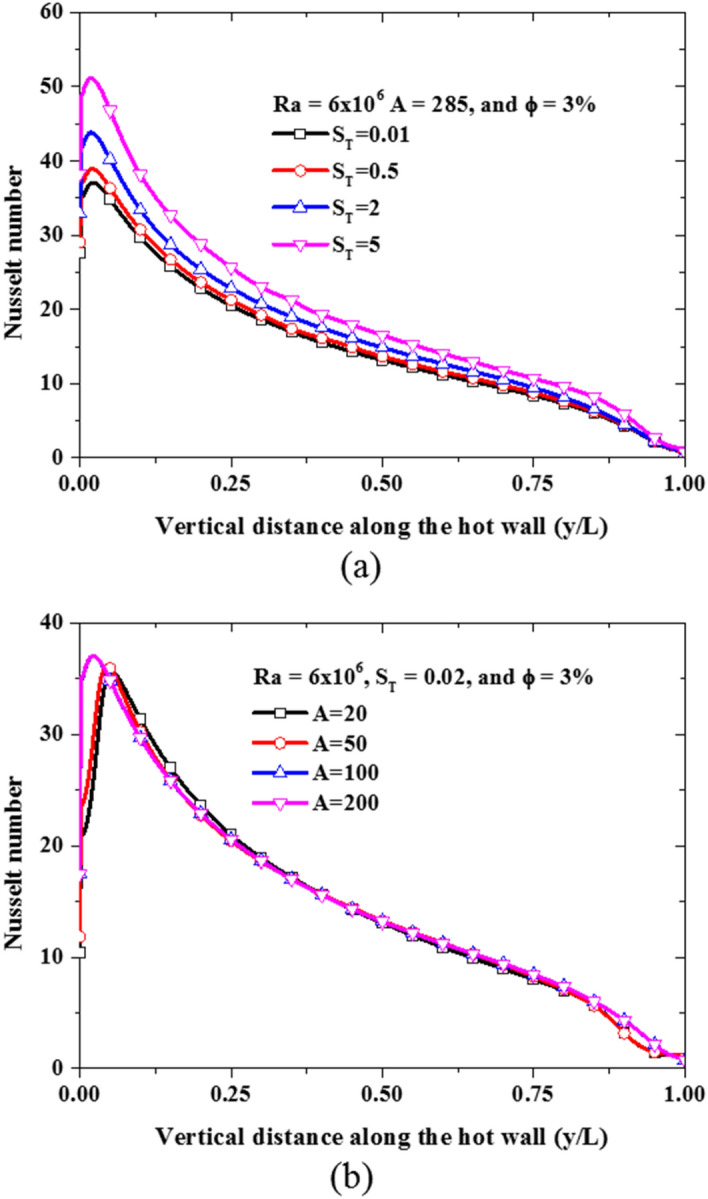
Local Nusselt number along the hot wall: **(a)** under the influence of thermophoretic diffusion, and **(b)** under the influence of sedimentation.

The impact of the non-uniformity of nanoparticle distribution caused by the various transport mechanisms on the local nanofluid heat transfer represented by the local Nusselt number is shown in Fig. 17. As the thermophoretic diffusion increases, the local Nusselt number curve of the hot and cold walls is shifted upward indicating enhancement of the nanofluid heat transfer as can be seen in Fig. 17a. On the contrary, the increase in sedimentation of nanoparticles due to gravity causes the local Nusselt number to decrease near the top and bottom wall as shown in Fig. 17b. This is because the effective conductivity of the nanofluid is reduced at the top while the effective viscosity increased at the bottom. This is due to the sharp reduction of nanoparticle concentration at the top (almost pure base fluid) and a large increase of nanoparticle concentration at the bottom. The corresponding effect of this variation of nanoparticle concentration at the top and bottom could be seen from the empirical correlations of the effective thermal conductivity and viscosity earlier shown in Table 2.

**Figure 18:**
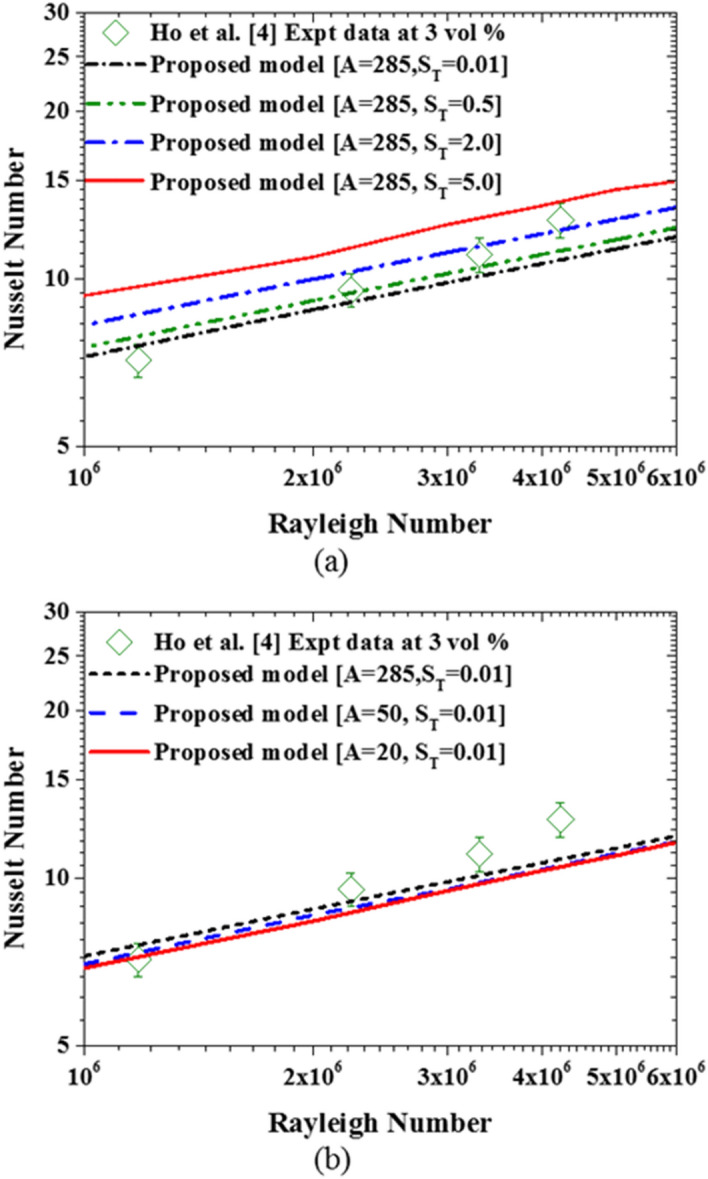
Impact of the slip mechanisms on the nanofluid heat transfer: **(a)** thermophoretic diffusion, **(b)** sedimentation of nanoparticles.

After ascertaining that the two dominant slip mechanisms have been properly captured by the proposed two-phase model, the impact of these slip motions on the nanofluid natural convection heat transfer is investigated next. As shown in Fig. 18a, as the thermophoretic diffusion increases while sedimentation of nanoparticles is suppressed, the nanofluid heat transfer is enhanced. This is so because the energy transport by the migrating nanoparticles from hot regions to cold regions generally favors the essence of natural convection heat transfer. Although thermophoresis also causes nanoparticle deposition on the bottom wall, the migration of nanoparticles from hot wall to cold wall is far stronger as previously shown in Fig. 16. This can also be corroborated by the work of Buongiorno^18^ in which thermophoretic diffusion alongside Brownian diffusion has been used to explain the abnormal enhancement of nanofluid heat transfer that cannot be captured by dynamic thermophysical properties. Conversely, as the sedimentation of nanoparticles increases, while the thermophoretic diffusion is suppressed, the nanofluid heat transfer deteriorates as can be seen in Fig. 18b. The reason for this has been previously discussed and illustrated in Fig. 17. Moreover, the sedimentation of nanoparticles is more strongly dependent on the size of the nanoparticles than the settling coefficient as shown in Eq. (11). Therefore, it can be said that the thermophoretic diffusion and sedimentation of nanoparticles have opposing effects on the nanofluid heat transfer. While thermophoretic diffusion enhances nanofluid heat transfer, the sedimentation of nanoparticles deteriorates it. However, there is a need for experimental investigation of these nanoparticle slip mechanisms to develop accurate and dynamic formulation for settling and thermophoretic coefficients.

Having observed the impact of both thermophoretic diffusion and sedimentation of particles, the two parameters ($A$ and ${S}_{T}$) appear to depend on nanoparticle concentration and Rayleigh number. Therefore, a generalized expression for the two parameters in terms of $\varphi$ and Ra are formulated and are written as shown in Eqs. (30) and (31).

30 $$A={a}_{1}+{a}_{2}\mathrm{ln}\left({\varphi }_{avg}\right)+{a}_{3}\mathrm{ln}\left(Ra\right)$$

31 $${S}_{T}=\left[{b}_{1}+{b}_{2}\mathrm{ln}\left({\varphi }_{avg}\right)+{b}_{3}\mathrm{ln}\left(Ra\right)\right]\frac{{k}_{m}}{2{k}_{m}+{k}_{p}}$$

where ${a}_{1}$, ${a}_{2}$, ${a}_{3}$, ${b}_{1}$, ${b}_{2}$, and ${b}_{3}$ are constants that can be determined through multiple regressions of data for $A$ and ${S}_{T}$ obtained using experimental measurements of nanofluid heat transfer as reference data. Using Ho et al*.*^4^ experimental data set for nanofluid concentrations of 1% and 3%, and Rayleigh number ranging from 1 × 10^6^ to 1 × 10^7^, the values of the unknown parameters $A$ and ${S}_{T}$ that minimize the standard deviation of errors between the predicted heat transfer data and experimental heat transfer data are determined. The generated values of these parameters are then used to determine the constants in Eqs. (30) and (31) through multiple regression analysis. Thus, the values of ${a}_{1}$, ${a}_{2}$, ${a}_{3}$, ${b}_{1}$, ${b}_{2}$, and ${b}_{3}$ computed are shown in Table 4.

**Table 4:** Correlation parameters for sedimentation and thermophoretic coefficients.

	Parameter		
Sedimentation coefficient	${a}_{1}=-1020$	${a}_{2}=168$	${a}_{3}=118$
Thermophoretic coefficient	${b}_{1}=-533.876$	${b}_{2}=66.04$	${b}_{3}=56.42$

**Figure 19:**
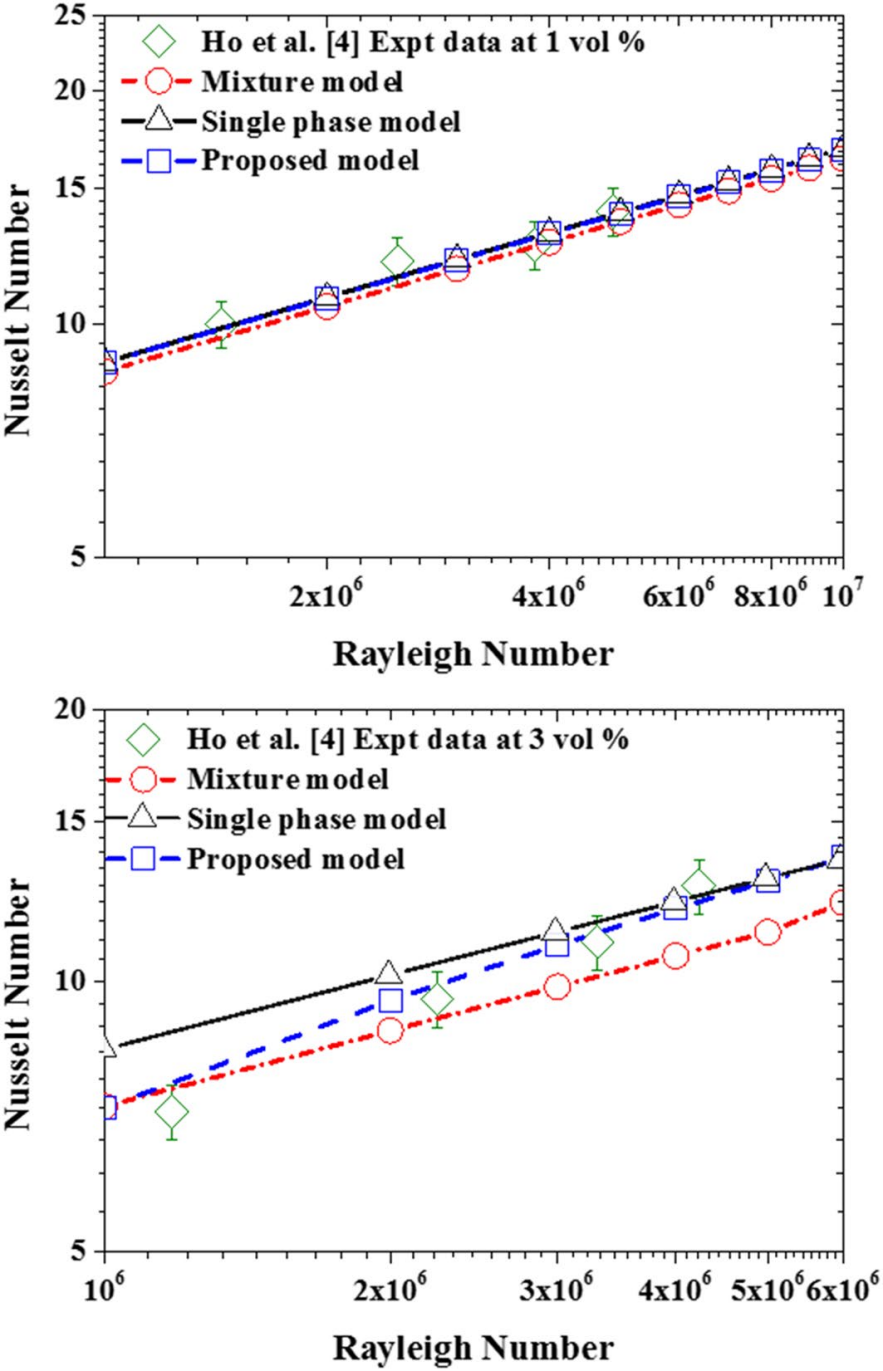
Impact of the calibrated sedimentation and thermophoretic parameters.

**Figure 20:**
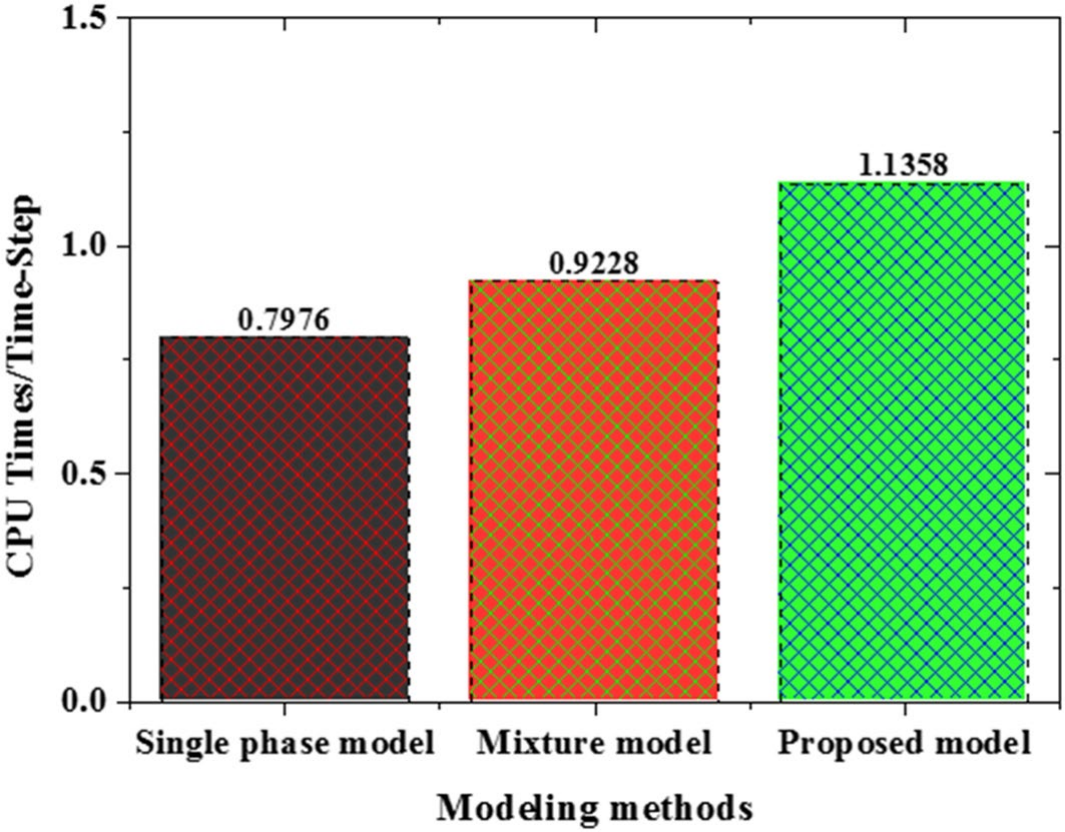
Computation cost of the three models.

Deploying Eqs. (30) and (31) in the proposed two-phase model yields a much better prediction of experimental data as shown in Fig. 19. However, further calibration of these two important parameters using a wider range of experimental data is required for a more accurate prediction of nanofluid heat transfer at high nanoparticle concentrations. Finally, as shown in Fig. 20, the associated computational cost of using this new two-phase model over the single-phase model is acceptable especially, considering the derivable benefits in terms of its accuracy.

## Conclusion

This work proposes an enhanced two-phase drift-flux nanofluid model, by accounting for the slip nanoparticles mechanisms: Brownian and thermophoresis diffusions and more importantly the nanoparticle sedimentation. Numerical experiments of buoyancy-driven nanofluid in a differentially heated square cavity showed that the proposed model can predict the heat transfer rates for a variety of nanoparticle void fractions (from 0 to 3%) and a wide range of Rayleigh numbers. It is worth noticing that assessments of existing single-phase and two-phase models against experimental data performed in this study have revealed the inadequacies of these models in obtaining the correct heat transfer rate magnitude. Their respective outcomes are seriously affected by the nanoparticle void fraction and Rayleigh number.

Above and beyond, as the thermophoresis diffusion enhances the heat transfer, the sedimentation of nanoparticles deteriorates it. The Brownian motion term, on the other hand, is found to have a negligibly small contribution. Considering this, an empirical correlation is proposed for the controlling parameters appearing in the thermophoresis diffusion and sedimentation terms (*A*, *S*_*T*_) and demonstrated better predictions of nanofluid heat transfer. Furthermore, the associated additional cost required to achieve this numerical prediction improvement remains within the acceptable range for industrial applications. Future work, planned by the team, will consist in further calibration of these empirical correlations using a wider range of experimental data and flow conditions.
